# Deglutarylation of glutaryl-CoA dehydrogenase by deacylating enzyme SIRT5 promotes lysine oxidation in mice

**DOI:** 10.1016/j.jbc.2022.101723

**Published:** 2022-02-12

**Authors:** Dhaval P. Bhatt, C. Allie Mills, Kristin A. Anderson, Bárbara J. Henriques, Tânia G. Lucas, Sara Francisco, Juan Liu, Olga R. Ilkayeva, Alexander E. Adams, Shreyas R. Kulkarni, Donald S. Backos, Michael B. Major, Paul A. Grimsrud, Cláudio M. Gomes, Matthew D. Hirschey

**Affiliations:** 1 Duke Molecular Physiology Institute, Duke University School of Medicine, Durham, North Carolina, USA; 2 Department of Cell Biology and Physiology, Washington University in St. Louis, St. Louis, Missouri, USA; 3 Sarah W. Stedman Nutrition and Metabolism Center, Duke University School of Medicine, Durham, North Carolina, USA; 4 Departments of Medicine and Pharmacology & Cancer Biology, Duke University School of Medicine, Durham, North Carolina, USA; 5 Biosystems and Integrative Sciences Institute, Faculdade de Ciências, Universidade de Lisboa, Lisboa, Portugal; 6 Departmento de Química e Bioquimica, Faculdade de Ciências, Universidade de Lisboa, Lisboa, Portugal; 7 Department of Medicine, Division of Endocrinology, Metabolism, and Nutrition, Duke University School of Medicine, Durham, North Carolina, USA; 8 Computational Chemistry and Biology Core Facility, Skaggs School of Pharmacy and Pharmaceutical Sciences, University of Colorado Anschutz Medical Campus, Aurora, Colorado, USA

**Keywords:** sirtuin, posttranslational modification (PTM), liver, amino acid, cell metabolism, sirtuin 5 (SIRT5), glutarylation, glutaryl-CoA dehydrogenase (GCDH), lysine metabolism, ACN, acetonitrile, AmBIC, ammonium bicarbonate, BN-PAGE, blue native polyacrylamide gel electrophoresis, CD, circular dichroism, DCPIP, 2,6-dichlorophenolindophenol, DMEM, Dulbecco’s modified Eagle’s medium, EBSS, Earle’s Balanced Salt Solution, ETF, electron transfer flavoprotein, FBS, fetal bovine serum, FDR, false discovery rate, GCDH, glutaryl-CoA dehydrogenase, IP, immunoprecipitation, PMS, phenazine methosulfate, PTM, posttranslational modification, RACS, reactive carbon species, SIRT5, sirtuin 5, TBS, tris-buffered saline

## Abstract

A wide range of protein acyl modifications has been identified on enzymes across various metabolic processes; however, the impact of these modifications remains poorly understood. Protein glutarylation is a recently identified modification that can be nonenzymatically driven by glutaryl-CoA. In mammalian systems, this unique metabolite is only produced in the lysine and tryptophan oxidative pathways. To better understand the biology of protein glutarylation, we studied the relationship between enzymes within the lysine/tryptophan catabolic pathways, protein glutarylation, and regulation by the deglutarylating enzyme sirtuin 5 (SIRT5). Here, we identify glutarylation on the lysine oxidation pathway enzyme glutaryl-CoA dehydrogenase (GCDH) and show increased GCDH glutarylation when glutaryl-CoA production is stimulated by lysine catabolism. Our data reveal that glutarylation of GCDH impacts its function, ultimately decreasing lysine oxidation. We also demonstrate the ability of SIRT5 to deglutarylate GCDH, restoring its enzymatic activity. Finally, metabolomic and bioinformatic analyses indicate an expanded role for SIRT5 in regulating amino acid metabolism. Together, these data support a feedback loop model within the lysine/tryptophan oxidation pathway in which glutaryl-CoA is produced, in turn inhibiting GCDH function *via* glutaryl modification of GCDH lysine residues and can be relieved by SIRT5 deacylation activity.

Protein posttranslational modifications (PTMs) are an evolutionarily conserved mechanism of cellular control across species (1, 2, 3). The discovery of several novel protein acyl modifications has expanded the spectrum of known PTMs in biology, including lysine propionylation, malonylation, butyrylation, beta-hydroxybutyrylation, succinylation, glutarylation, hydroxymethylglutarylation, methylglutarylation, methylglutaconylation, crotonylation, myristoylation, and palmitoylation [see (1, 2, 4) for reviews]. Large-scale proteomic studies have identified several biological targets of protein acylation, enhancing our understanding of the mechanisms and biological conditions leading to these acyl modifications (4, 5, 6).

Protein acetylation and acylation in the nucleus and cytoplasm are primarily facilitated by acyltransferase enzymes; however, under conditions of elevated pH, such as in the mitochondrial environment, enzyme acylation can occur nonenzymatically. Thus far, in the absence of *bona fide* mitochondria acyltransferases, the primary mechanism for mitochondrial protein acylation is generally considered to be nonenzymatic. Metabolite-based reactive carbon species (RACS) are often generated as metabolic intermediates, with a number of PTMs corresponding to their cognate acyl-CoA species.

While protein modification *via* RACS is emerging as nonenzymatic, acylation removal is enzymatically catalyzed by sirtuin protein deacylases. Sirtuins are a class of enzymes associated with stress response and aging (7, 8). The mitochondrial sirtuins, SIRT3, SIRT4, and SIRT5, have an expanding repertoire of deacylase activities; however, the biological roles of the mitochondrial sirtuins, and the acyl modifications they regulate, remain unclear.

We previously showed that mice lacking SIRT5 have hyperglutarylated mitochondrial proteins (9), which play a key role in regulating the enzyme CPS1 in ammonia detoxification and the urea cycle (10). The only known source of glutarylation is glutaryl-CoA, a 5-carbon metabolite exclusively produced in the lysine (KEGG: hsa00310)/tryptophan (KEGG: map00380) catabolic pathways in mammalian systems (11). Furthermore, we previously identified glutaryl-CoA as a reactive carbon species (4), thus we predicted that SIRT5-mediated removal of protein glutarylation might control enzymes activity in the glutaryl-CoA metabolism pathway. Thus, we set out to test this hypothesis.

## Results

Because of the emerging idea that proteins in the vicinity of reactive acyl-CoA’s are susceptible to nonenzymatic acylation of lysine residues, we explored protein glutarylation in the lysine/tryptophan degradation pathways. Within these pathways, α-ketoadipate is converted to glutaryl-CoA by a protein complex involving dehydrogenase E1 and transketolase domain containing 1 and components of the 2-oxoglutarate dehydrogenase complex including dihydrolipoyllysine-residue succinyltransferase/dihydrolipoyl dehydrogenase. However, none of these protein complex components are unique to the lysine/tryptophan degradation pathways, which might obfuscate testing this hypothesis. Further downstream in this degradation pathway, glutaryl-CoA is converted into crotonyl-CoA by glutaryl-CoA dehydrogenase (GCDH), which is exclusive to the lysine/tryptophan degradation pathways. Therefore, we selected GCDH as a putative target protein for further testing.

**Figure 1:**
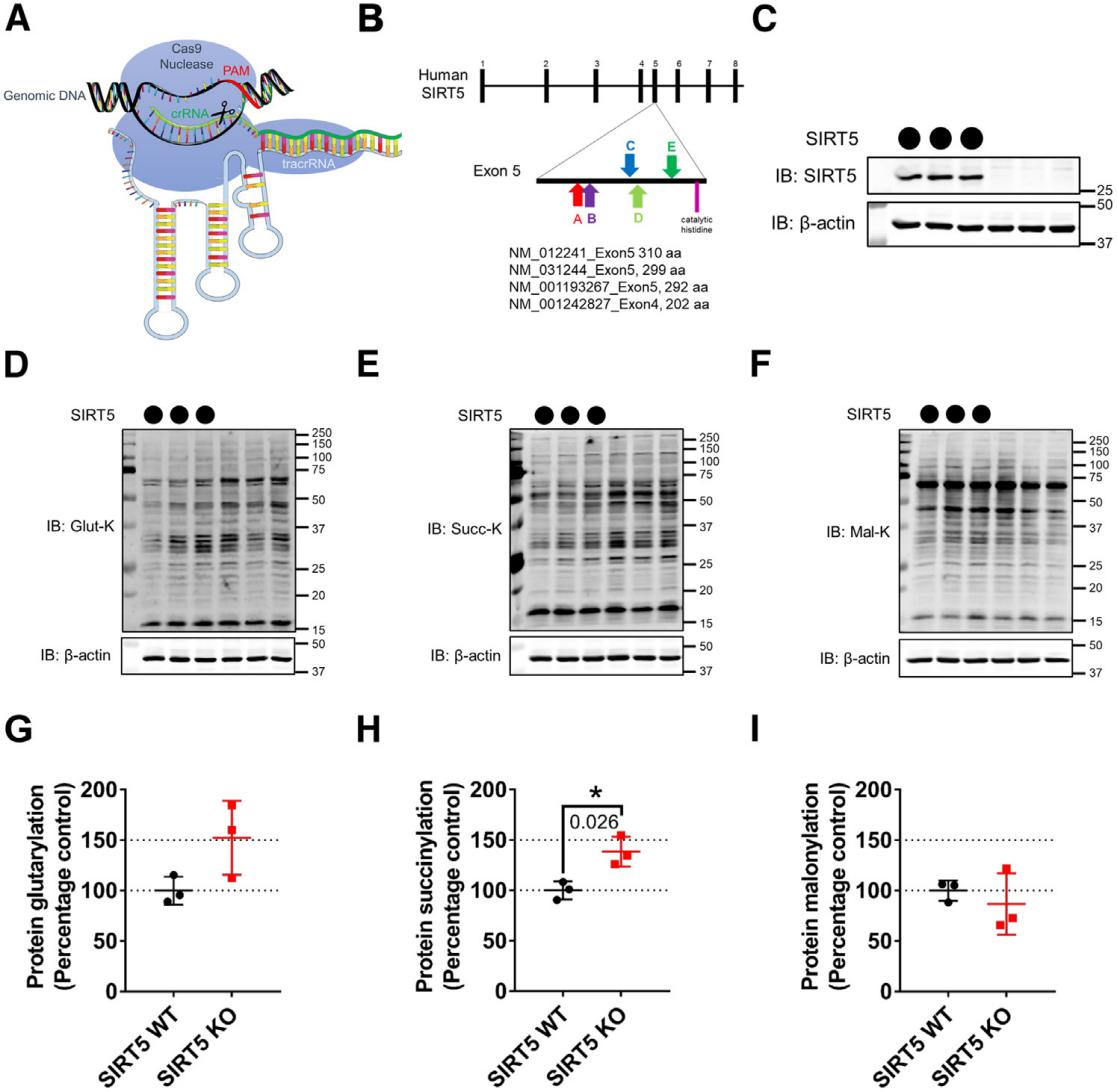
**Characterization of the SIRT5 crKO cell line.***A*, schematic of the CRISPR-Cas9 system used to generate the SIRT5 crKO cell line. *B*, schematic of the human *SIRT5* gene showing the sites of crRNA targets within Exon5/4 the four known SIRT5 coding transcripts in humans. *C*, immunoblot for SIRT5 protein from HEK293T crWT (*circles*) clone and crKO (*blank*) clone (using CRISPR guide E, Table S1). *D*–*F*, immunoblots of SIRT5 crWT and crKO whole-cell lysates blotted using antibodies against glutaryl-lysine (*D*), glutaryl-succinyl-lysine (*E*), and malonyl-lysine (*F*). *G*–*I*, relative quantification of acylated proteins normalized to β-actin levels: glutarylation (*G*), succinylation (*H*), and malonylation (*I*) (mean ± SD, n = 3, ∗*p*-value ≤ 0.05). crRNA, crispr RNA; PAM, protospacer adjacent motif; SIRT5, sirtuin 5; tracrRNA, trans-activating crispr RNA.

Of all the known sirtuin activities, SIRT5 is the only one known to possess strong deglutarylase activity (9), in addition to its desuccinylase and demalonylase activities (12, 13, 14). To test how SIRT5 influenced the acylation of GCDH, we generated a *Sirt5*^−*/*−^ (SIRT5KO) cell line using the CRISPR-Cas9 system (Fig. 1*A*) in HEK293 T cells (SIRT5 crKO). Five different crRNA’s (Fig. 1*B*) targeting different regions upstream of the catalytic histidine in the exon 4 or 5 of the four known coding transcripts of *SIRT5* generated 75 monoclonal colonies. The control cell line (SIRT5 crWT) was processed through the same CRISPR-Cas9 workflow like the SIRT5 crKO cells except the *SIRT5* crRNA was omitted. We screened each colony for SIRT5 protein levels using immunoblot and found one colony (cr guide E, Fig. 1*B* and Table S1) with complete SIRT5 depletion (Figs. 1*C* and S1, *A* and *B*). Surprisingly, during screening, we noticed some colonies showing ∼33% to 66% reduction of SIRT5 protein. Upon examining the HEK293T karyotyping and copy number analysis (15), it became clear that these hypotriploid cells have three copies of the human *SIRT5* gene. Using immunoblots, we monitored the SIRT5 crKO clone for SIRT5 protein levels over several passages and checked for changes in protein acylation. While no SIRT5 protein was detected in the SIRT5 crKO cell line for at least 15 passages (data not shown), we found modest increases in global protein glutarylation and succinylation but not malonylation or acetylation relative to the control SIRT5 crWT line (passage 10, Figs. 1, *D*−*I* and S1, *C* and *D*). Thus, this SIRT5 crKO cell line appeared to be a suitable model to study the role of SIRT5 and protein glutarylation.

### GCDH is hyperglutarylated in the absence of SIRT5

**Figure 2:**
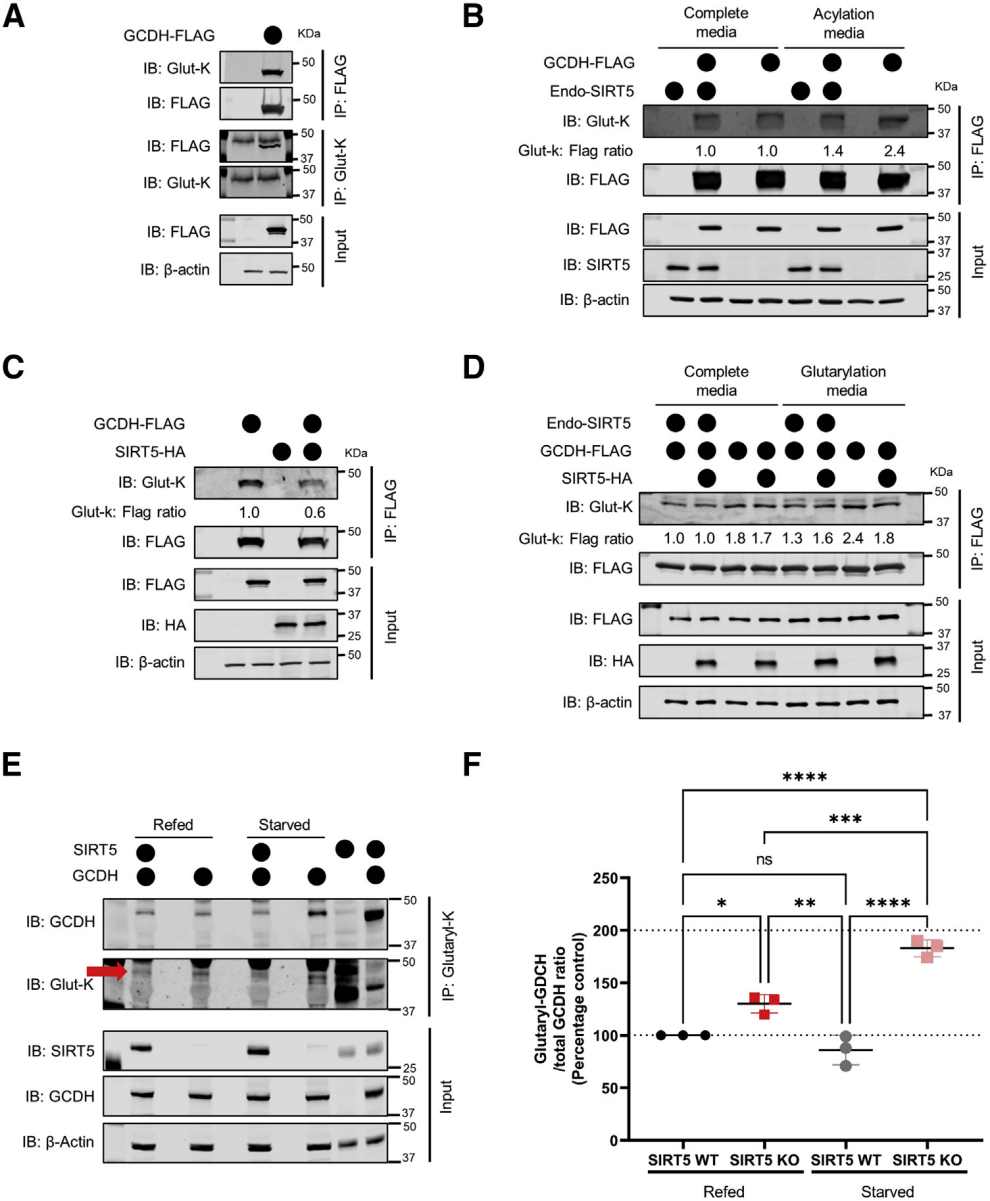
**Glutarylation-state of GCDH is regulated by Sirt5.***A*, immunoblot for immunopurified GCDH-FLAG by Flag-M2 resin or glutaryl-proteins by anti-glutaryl-lysine antibody (Glut-K) from HEK293T cells grown in complete media. Blots representative of at least three independent experiments. *B*, immunoblot for immunopurified GCDH-FLAG by Flag-M2 resin from SIRT5 crWT or crKO cells grown in complete media or acylation media (DMEM without glucose, glutamine, pyruvate, 10% FBS). Blots are representative of at least three independent experiments and quantitative values of glutaryl-lysine intensity normalized to FLAG intensity expressed as ratios relative to control (overexpressed GCDH-FLAG in SIRT5 crWT cells grown in complete media, lane 2). *C*, immunoblot of immunopurified GCDH-FLAG by Flag-M2 resin from HEK293T cells grown in complete media with or without coexpressed SIRT5-HA. Blots representative of at least three independent experiments and quantitative values of glutaryl-lysine intensity normalized to FLAG intensity expressed as ratios relative to control (overexpressed GCDH-FLAG, lane 2). *D*, immunoblot for immunopurified GCDH-FLAG by Flag-M2 resin from SIRT5 crWT or crKO cells grown in complete media or glutarylation media (EBSS containing 5 mM glucose, +50 mM Hepes, +0.8 mM lysine) with or without coexpressed SIRT5-HA. Blots are representative of at least three independent experiments and quantitative values of glutaryl-lysine intensity normalized to FLAG intensity expressed as ratios relative to control (overexpressed GCDH-FLAG in SIRT5 crWT cells grown in complete media, lane 1). *E*, immunoblot for pan-glutaryl lysine immunopurified proteins by endogenous GCDH and glutaryl-lysine from 3-month-old wild-type (SIRT5WT) and *Sirt5^−/−^* (SIRT5KO) mice liver tissue that were refed or starved for 1 h following a 24 h starvation. *Red arrow* indicates the GCDH band within the glutaryl blot. Blots representative of at least three mice per group (n = 3 per group). *F*, relative quantification of the immunoblots in (*E*). Data representative of a single experiment from six SIRT5WT and six SIRT5KO mouse livers starved for 24 h from which half were refed (3 SIRT5WT and 3 SIRT5KO) normal diet for 1 h, while the remaining half (three SIRT5WT and three SIRT5KO) continued starvation for additional 1 h (mean ± SD, n = 3 per group). WT and *Gcdh^−/−^* (GCDHKO) mice (n = 1) were used as positive and negative controls, respectively, for identifying the correct GCDH band. One-way ANOVA followed by Tukey’s post hoc test was performed for multiple comparisons across groups where ∗*p*-value = 0.0173, ∗∗*p*-value = 0.0018, ∗∗∗*p*-value = 0.0005, ∗∗∗∗*p*-value < 0.0001. DMEM, Dulbecco’s modified Eagle’s medium; EBSS, Earle's Balanced Salt Solution; endo-SIRT5, endogenous SIRT5; FBS, fetal bovine serum; GCDH, glutaryl-CoA dehydrogenase.

To determine the acylation state of GCDH, we first overexpressed a C-terminal FLAG-tagged human GCDH protein (GCDH-FLAG) in HEK293T cells under basal cell culture conditions (high-glucose Dulbecco’s modified Eagle’s medium [DMEM], containing 4 mM glutamine, and 10% fetal bovine serum [FBS]). Overexpressed GCDH-FLAG was immunoprecipitated using a FLAG-M2 antibody-conjugated resin and probed for glutaryl-lysine using immunoblot. We found that overexpressed GCDH-FLAG was glutarylated (Fig. 2*A*). To confirm this finding, we performed a reverse immunoprecipitation experiment. Glutarylated proteins were immunoprecipitated using an anti–glutaryl-lysine antibody and analyzed using immunoblot analysis with anti-FLAG antibody. We found a distinct band detecting the FLAG tag on overexpressed GCDH-FLAG at ∼43 kDa (Fig. 2*A*), the molecular weight of mitochondrial GCDH. These data provide evidence that GCDH is acylated.

Next, we overexpressed GCDH-FLAG in SIRT5 crKO cells, predicting that the absence of SIRT5 would lead to an increase in glutarylation of GCDH. However, we did not find any significant change in the glutarylation state of overexpressed GCDH-FLAG in SIRT5 crKO cells under basal cell culture conditions (Fig. 2*B*). Since cells were grown in complete media with abundant nutrients, we hypothesized the cells might not be metabolizing lysine/tryptophan; the only known pathways that generate glutaryl-CoA. Therefore, we reasoned that under basal cell growth conditions, glutaryl-CoA levels may not be produced in quantities high enough to induce hyperglutarylation. We repeated the same experiment in cell culture media designed to drive catabolism of amino acids and ultimately acylation, here termed acylation media (DMEM without glucose, glutamine, pyruvate, and serum). We assessed the glutarylation state of overexpressed GCDH-FLAG under these media conditions where the only oxidative fuels available to these cells were amino acids (excluding glutamine), which drive acyl-CoA formation resulting in increased acylation. We found that driving amino acid oxidation using this acylation media increased glutarylation of GCDH-FLAG in both SIRT5 crWT and crKO cells relative to cells grown in complete media, however to a much higher degree in the SIRT5 crKO cells (2.4-fold) compared to the SIRT5 crWT cells (1.4-fold; Fig. 2*B*). Therefore, these data suggest that limiting the availability of nonamino acid fuel sources increases glutarylation of GCDH in SIRT5 crWT cells, which further increases in the absence of the protein deacylase SIRT5.

To determine the specificity of this model to glutaryl-CoA, we tested whether succinylation, another modification targeted by SIRT5, modifies GCDH under conditions similar to glutarylation. FLAG-lysine and succinyl-lysine immunoprecipitation experiments confirmed that overexpressed GCDH-FLAG was succinylated (Fig. S2*A*). We further investigated whether the absence of SIRT5 altered the succinylation state of overexpressed GCDH-FLAG using identical conditions tested for glutarylation in SIRT5 crWT and SIRT5 crKO with complete and acylation media. Unlike glutaryl-GCDH, we found that under basal conditions, GCDH-FLAG was hypersuccinylated in SIRT5 crKO cells (Fig. S2*B*). Our previous work has shown that succinyl-CoA is more reactive than glutaryl-CoA, as it has a greater ability (approximately 10-fold) to form a cyclic anhydride (4). Supporting these data, succinyl-CoA–modified proteins show greater molecular weight shifts (4) compared to glutaryl-CoA or any other 4-5 carbon dicarboxylic acid. Thus, basal hypersuccinylation of GCDH-FLAG could be due to elevated reactivity of succinyl-CoA and/or its higher cellular abundance. Moreover, similar to glutaryl-GCDH, the acylation medium further increased succinylation of GCDH-FLAG in both SIRT5 crWT and crKO cells, again to a greater extent in the SIRT5 crKO cells (3.5-fold) than the SIRT5 crWT cells (1.7-fold) (Fig. S2*B*).

The acylation medium used in these experiments had normal levels of all amino acids except glutamine. Under these conditions, in the absence of preferred fuel sources (glucose, glutamine, and pyruvate), cells are forced to metabolize amino acids for energy production. Strikingly, both succinyl-CoA-generating (derived from valine and isoleucine) and glutaryl-CoA-generating (derived from lysine and tryptophan) amino acids were available to the cells and could potentially explain the increase in succinyl and glutarylmodifications of GCDH in amino acid catabolic conditions.

### SIRT5 deglutarylates GCDH

To further test the relationship between GCDH and SIRT5-mediated deglutarylation, we co-overexpressed GCDH-FLAG and a C-terminal HA-tagged human SIRT5 in HEK293 T cells. GCDH-FLAG affinity-purified from cells overexpressing SIRT5-HA showed significantly less (0.6-fold) glutarylation compared to that from cells expressing endogenous levels of SIRT5 (Fig. 2*C*). Interestingly, while testing the ability of overexpressed SIRT5 to desuccinylate GCDH-FLAG, we found that, unlike glutaryl-GCDH, coexpression with SIRT5-HA did not reduce the succinylation state of GCDH-FLAG (Fig. S2*C*). These data suggest that SIRT5 targets glutaryl-GCDH for deglutarylation but does not appear to alter the succinylation-state of GCDH as measured by immunoblotting.

To interrogate the specificity of SIRT5 toward glutaryl-GCDH, we performed GCDH-FLAG and SIRT5-HA overexpression in SIRT5 crWT and crKO cells in complete and glutarylation-promoting conditions. To do so, we created a glutarylation media composed of Earle’s Balanced Salt Solution (EBSS containing 5 mM glucose) supplemented with 50 mM Hepes and 0.8 mM lysine (as a source of glutaryl-CoA). Our rationale was to grow cells in a medium with minimal fuel sources to drive lysine oxidation and generate the substrate for glutarylation (glutaryl-CoA) alone, rather than in a medium that generates precursors for both glutarylation and succinylation (succinyl-CoA), as occurs in the abovementioned acylation-promoting medium. Interestingly, we found SIRT5-HA was only able to deglutarylate GCDH-FLAG under these nutrient deprivation conditions in the absence of endogenous SIRT5 (SIRT5 crKO; Fig. 2*D*). As expected, the increase in succinylation of GCDH in glutarylation media was not as pronounced as in acylation-promoting media (Fig. S2, *B versus D*). Absence of succinyl-CoA generating amino acids in the glutarylation media may explain the differences in succinylation of GCDH-FLAG compared to the marked increase (∼3.5 fold) observed in the acylation media (Fig. S2, *B versus D*). However, consistent with the coexpression of SIRT5 and GCDH in HEK293T cells (Fig. S2*C*), overexpressed SIRT5 modestly desuccinylated GCDH-FLAG (Fig. S2*D*). Overall, these data support a role for SIRT5 in deglutarylating GCDH, but not desuccinylating the protein under nutrient deprivation conditions.

To further explore this regulation *in vivo*, we collected liver tissue from 3-month-old SIRT5WT and SIRT5KO mice that were starved/deprived of food for 24 h followed by 1 h of free access to food (Refed condition, n = 3 each for SIRT5WT and SIRT5KO) or continued starvation for additional 1 h (Starved condition, n = 3 each for SIRT5WT and SIRT5KO). We immunopurified glutarylated proteins using the PTMScan pan-glutaryl-lysine affinity resin (Cell Signaling Technologies) and immunoblotted for endogenous GCDH. Immunoblots in Figure 2*E*, quantified in Figure 2*F*, clearly showed significantly more glutaryl-GCDH in the starved SIRT5KO mouse liver in the starved or refed state compared to either the starved SIRT5WT or refed SIRT5KO mouse liver (Fig. 2, *E* and *F*). It is important to note that while more GCDH was immunopurified in the starved SIRT5KO mouse livers compared to other groups, the total normalized glutarylation level of GCDH was ∼2-fold higher in the starved SIRT5KO group than other groups (Fig. 2, *E* and *F*.). Therefore, these data in combination with data from the cellular model validate the physiological role of SIRT5 in regulating the glutarylation state of GCDH.

### Glutarylation's impact on GCDH activity

**Figure 3:**
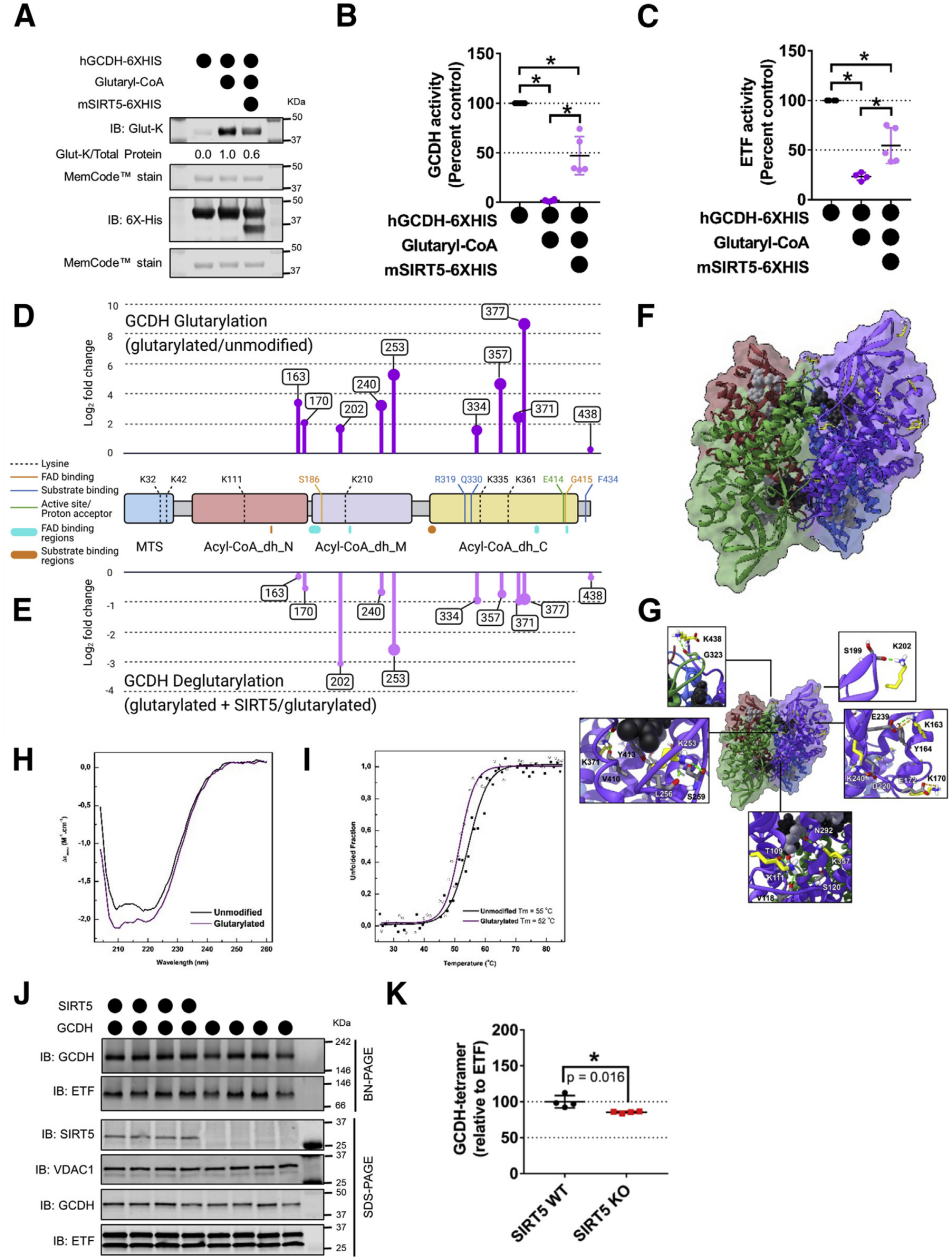
**Glutarylation impairs GCDH activity and SIRT5 partially restores it.***A*, immunoblot of chemically modified GCDH (hGCDH-6xHIS) using glutaryl-CoA with or without incubation with recombinant SIRT5 (mSIRT5-6xHIS). Quantitative values of glutaryl-lysine intensity normalized to total protein are expressed as ratios relative to control (glutaryl-modified recombinant GCDH, lane 2). *B*, enzymatic activity of unmodified (*black*), glutarylated (*dark purple*), and deglutarylated (*light purple*) GCDH determined by using PMS/DCPIP as artificial electron acceptors and glutaryl-CoA as electron donor (mean ± SD, n = 6, 6, and 5 for unmodified, modified, and deacylated GCDH respectively; ∗*p*-value ≤ 0.05). *C*, enzymatic activity of unmodified (*black*), glutarylated (*dark purple*), and de-glutarylated (*light purple*) GCDH determined by using ETF/DCPIP as an electron acceptor and glutaryl-CoA as electron donor (mean ± SD, n = 4, 4, and 5 for unmodified, modified, and deacylated GCDH respectively, ∗*p*-value ≤ 0.05). *D* and *E*, glutaryl-lysine sites identified by label-free quantitative LC-MS/MS on recombinant GCDH mapped to full-length human GCDH protein schematic with domain and residues important for cofactor, substrate, or tetramer interactions. Panel *D* shows glutaryl-lysine sites in chemically glutarylated GCDH sample expressed as a ratio over unmodified GCDH (glutarylation: glutarylated GCDH/unmodified). Panel *E* shows glutaryl-lysine sites on chemically glutarylated GCDH altered by SIRT5 incubation expressed as a ratio over glutarylated GCDH (deglutarylation: (glutarylated GCDH + SIRT5)/glutarylated GCDH). Plots representing Log_2_ fold change (y-axis) and *p*-value (larger circle size = more significant *p*-value). *F* and *G*, homotetrameric structure of human GCDH with each subunit colored separately (*purple*, *blue*, *red*, and *green*). *Yellow sticks* indicate modified lysine residues. The FAD cofactor and CoA substrate are *black* and *light gray spheres*, respectively. *H*, far-UV CD spectra for glutarylated GCDH (*purple*) and unmodified GCDH (*black*) corrected for protein concentration (5 μM). *I*, thermal stability profiles for glutarylated GCDH (*open circles*, *purple curve*) and unmodified GCDH (*closed squares*, *dark curve*). The solid lines represent two-state sigmoid curves from which the apparent midpoint temperatures were determined. *J*, immunoblot of native-PAGE separated GCDH tetramer and SDS-PAGE for denatured GCDH isolated from liver mitochondria of 24 h starved SIRT5WT and SIRT5KO mice. *K*, quantitation of GCDH tetramer intensity normalized to ETF complex intensity. Data representative of two independent experiments (mean ± SD, n = 3 for both SIRT5WT and SIRT5KO). ETF, electron transfer flavoprotein; GCDH, glutaryl-CoA dehydrogenase; SIRT5, sirtuin 5.

To understand the impact of glutarylation and the role of SIRT5 in regulating GCDH activity, we replicated our cell culture model *in vitro* through chemical acylation of human recombinant GCDH protein (hGCDH-6XHIS) using excess acyl-CoA and performed *in vitro* deacylation assays by coincubation with mouse recombinant SIRT5 (mSIRT5-6XHIS). Remarkably, immunoblot analysis of chemically acylated and/or deacylated GCDH showed a pattern similar to our cellular model described in Figure 2, *E* and *F*. Compared to maximally glutarylated GCDH, addition of SIRT5 reduced GCDH glutarylation ∼40% (Fig. 3*A*). However, the presence of SIRT5 resulted in only a modest (∼10%) decrease in GCDH succinylation (Fig. S3*A*). These data further support our findings that SIRT5 preferentially targets glutaryl-lysine modification on GCDH over succinyl-lysine.

Taking advantage of this pure human GCDH protein in the chemical acylation and *in vitro* deacylation system, we measured the specific activity of GCDH to elucidate the impact of acylation on enzyme function. For this *in vitro* assay, we determined the ability of acylated-recombinant and deacylated-recombinant GCDH to transfer electrons from glutaryl-CoA oxidation to either an artificial electron acceptor, phenazine methosulfate (PMS), or its endogenous electron acceptor, electron transfer flavoprotein (ETF). Interestingly, we found that chemically glutarylated GCDH had almost undetectable activity using the artificial electron acceptor PMS and about 20% residual activity with ETF when compared to unmodified GCDH (Fig. 3, *B* and *C*), both clearly demonstrating that glutarylation potently inhibits GCDH function. Incubation of modified GCDH with recombinant SIRT5 partially restored (∼50%) GCDH function (Fig. 3, *B* and *C*) in both conditions. Consistently, succinyl-GCDH also reduced GCDH activity to about 10% of unmodified GCDH, and incubation with SIRT5 was able to restore it to ∼30% (Fig. S3, *B* and *C*). These results are consistent with our cellular deacylation experiments, which suggest that although SIRT5 can target both glutaryl-lysine and succinyl-lysine sites on GCDH, it has a greater impact on deglutarylating GCDH. Furthermore, GCDH de-acylation by SIRT5 modulates GCDH activity.

To identify the sites of acylation/deacylation on GCDH, we turned to a well-established mass spectrometry–based proteomic approach using chemically modified and unmodified recombinant GCDH. GCDH protein was separated by SDS-PAGE, in-gel digested using trypsin, and analyzed *via* mass spectrometry. Glutaryl-GCDH or succinyl-GCDH peptide relative abundance was measured and normalized to GCDH peptides measured from unmodified protein. Using this strategy, we obtained 99% sequence coverage of GCDH protein, and all lysine residues corresponding to the cleaved mitochondrial protein were identified with acyl modifications (Table S2). Recombinant GCDH protein used for this analysis lacked the mitochondrial localization sequence; however, all residue locations referred in the figures and text correspond to the full-length human GCDH protein. Overall, we found 10 glutaryl-lysine (Fig. 3*D*) and 11 succinyl-lysine (Fig. S3*D*) sites on GCDH. Nine lysine residues were significantly glutarylated, with >1.8 Log_2_ fold-change above unmodified GCDH (*p* ≤ 0.05). Of these glutarylated sites, six were significantly deglutarylated (*p* ≤ 0.05; Fig. 3*C*), with lysine 253 (K253) having the most statistically significant reduction (2.7-fold change) compared to glutarylated GCDH. While the magnitude of fold change in succinylation of GCDH lysines (Fig. S3*D*) was observed by mass spectrometry, our immunoblot analysis suggested fewer sites are targeted by SIRT5 for desuccinylation (Fig. S3*A*). These data further support the notion that glutarylation of GCDH can be regulated by SIRT5.

To gain insight into how glutarylation influences GCDH conformation, we performed molecular modeling combined with a battery of biophysical measurements. GCDH functions as a homotetramer with the substrate (glutaryl-CoA, light gray spheres) and cofactor (FAD, black spheres) binding pockets (Figs. 3, *F* and *G* and S3, *E* and *F*). Modeling of acyl-lysine residues (Figs. 3*D* and S3*D*) on the crystal structure of human GCDH (PDB ID: 1SIR) suggests several of these sites can be important for the GCDH tetramer stability (K253, K335, K361, and K377), glutaryl-CoA substrate binding (K111, K163, K170, K210, K357, K361, K371, and K438), and FAD cofactor binding (K163, K170, K210, K253, K335, and K371). Notably, all heavily glutarylated lysine residues (163, 170, 253, 357, 371, and 377), except K202 and K240, have a strong potential to influence FAD/glutaryl-CoA binding and tetramer formation.

K163 and K253 both form important bonds involved in GCDH-FAD cofactor binding, and modification of these residues could impact this association (Fig. 3*G*). Furthermore, K253 and K377 both reside at the interface of adjacent GCDH monomers within the GCDH tetramer, and acylation of these residues can potentially influence tetramer stability (Figs. 3*G* and S3*F*). Additionally, K163, K170, and K240 are all located in proximity and involved in salt bridge interactions that are likely important for the overall cohesiveness of the protein structure. While a single modification at one of these locations may have a limited effect on protein structure, modification of all three simultaneously could reduce the overall stability of the protein structure and could indirectly affect substrate binding as this region is on the opposite side of the protein from the binding pocket (Fig. S3*G*).

To test the structural impact of acylation on the GCDH enzyme as suggested by molecular modeling, we performed extensive conformational and stability studies in purified heterologous expressed human GCDH (see Experimental procedures for details) using different spectroscopic techniques which provide information on different levels of protein structure. To assess the effect on GCDH’s secondary structure, we used circular dichroism (CD) in the far-UV region. We found that glutarylation results in a small decrease in ellipticity and a change in the ratio of the CD signals at 220 nm/208 nm, which is >1 in the unmodified enzyme and <1 in glutarylated GCDH, suggesting a slight change in the secondary structure content (Fig. 3*H*). In agreement, we observe a slight destabilization of the glutarylated protein, when monitoring thermal unfolding following changes in the secondary structure (ellipticity at 222 nm). We measured a slight decrease (ΔT_m_ = −3 °C) in the thermal stability of glutarylated GCDH compared to that of unmodified GCDH, which has a melting temperature (T_m_) of 55 °C ±1 deg. C (Fig. 3*I*). Parallel studies using succinylated GCDH showed a similar profile: a slight change in secondary structure content and decreased stability for modified protein (Fig. S3, *G* and *H*). We also monitored modified GCDH using fluorescence tryptophan emission to monitor tertiary structure and FAD emission to assess the cofactor environment. These studies showed no relevant alteration on protein conformation, as judged by spectra at 25 °C, or on protein thermal stability, as evaluated by thermal denaturation curves, irrespective of modifications (data not shown).

Furthermore, glutarylation abrogates the +1 charge present on lysine sidechains, leaving a −1 charged glutaryl modification, which is sterically larger than unmodified lysine. These changes in the steric space, lysine charge, or both, could disrupt interactions between protein subunits or protein–protein interactions. Since functional GCDH occurs as a homotetramer, we investigated whether altered steric and/or charged states could influence the native tetramer complex of GCDH. To do so, we isolated mitochondria from SIRT5WT and SIRT5KO mouse livers and performed blue native polyacrylamide gel electrophoresis (BN-PAGE) to monitor the GCDH tetramer. Identity of the correct GCDH tetramer band was confirmed by using GCDHWT and *Gcdh^−/−^* (GCDHKO) mouse liver mitochondrial lysates in parallel (Fig. S3*I*). In the fed state, we did not observe any significant change in the amount of the GCDH tetramer between the two genotypes (Fig. S3*I* and quantified Fig. S3*J*). Indeed, this agrees with size-exclusion chromatography assays performed with purified GCDH (unmodified and modified) which showed no difference in protein quaternary structure (data not shown). Since our previous analysis showed that acylation of GCDH increases during starvation, we isolated liver mitochondria from 24 h fasted SIRT5WT and KO mice and repeated the BN-PAGE analysis. We observed a modest but statistically significant decrease in the amount of GCDH tetramer in SIRT5KO *versus* SIRT5WT mouse liver mitochondria after fasting (Fig. 3*J* and quantified Fig. 3*K*). Therefore, while structural modeling suggests that tetramer formation or cofactor biding could be influenced by acylation, *in vitro* and *ex vivo* analysis showed that glutarylation has a subtle impact on GCDH folding.

### Lack of SIRT5 impairs glutaryl-CoA and lysine oxidation

**Figure 4:**
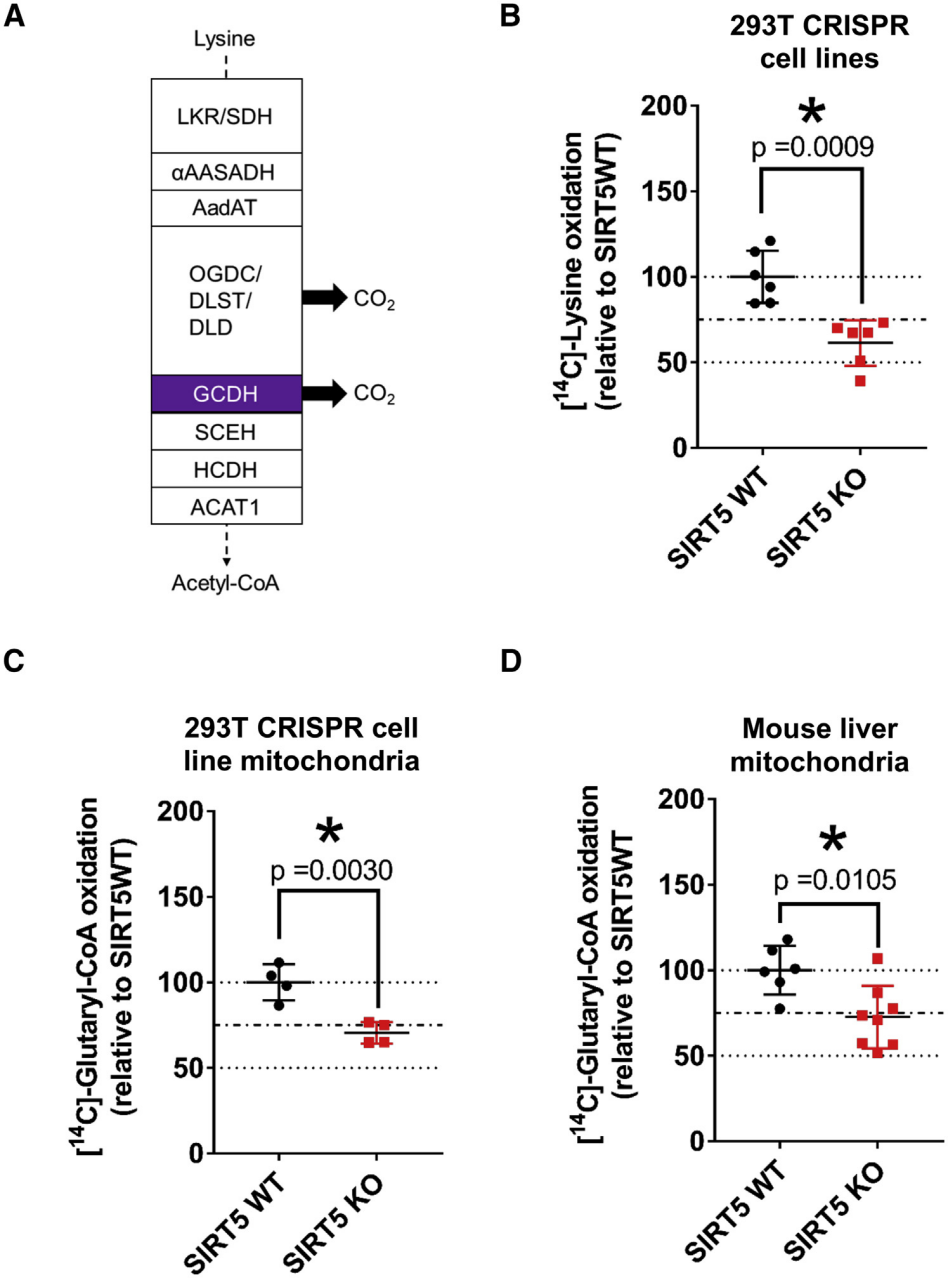
**SIRT5 ablation impairs lysine oxidation and glutaryl-CoA oxidation in cells and mice.***A*, schematic of lysine oxidation enzymes with steps that release carbon dioxide (CO_2_). *B*, oxidation of [U-^14^C]-lysine to [^14^C]-CO_2_ in 293T SIRT5 crWT and crKO cells. Data representative of three independent experiments (mean ± SD, n = 6; ∗*p*-value ≤ 0.05). *C*, oxidation of [1, 5-^14^C]-glutaryl-CoA to [^14^C]-CO_2_ in mitochondria isolated from 3 h starved 293T SIRT5 crWT and crKO cells. Data representative of single experiment (mean ± SD, n = 4; ∗*p*-value ≤ 0.05). *D*, oxidation of [1, 5-^14^C]-glutaryl-CoA to [^14^C]-CO_2_ in mitochondria isolated from SIRT5WT and SIRT5KO mouse liver. Data representative of single experiment (mean ± SD, n = 6, 8 for SIRT5WT and SIRT5KO respectively; ∗*p*-value ≤ 0.05). SIRT5, sirtuin 5.

As described above, GCDH is an enzyme involved in the lysine and tryptophan oxidation pathways. Lysine is one of the most abundant amino acids found in the body that can be used for ketogenesis during periods of starvation (16). During starvation, lysine is metabolized to acetyl-CoA and CO_2_ (Fig. 4*A*), which can be used to fuel the TCA cycle or used for other metabolic processes. Thus, we tested the impact of SIRT5 loss on lysine catabolism. First, we investigated the significance of hyperacylated GCDH on lysine oxidation. To do so, we modified an *in vitro* leucine oxidation assay (17) for measuring lysine metabolism using U-[^14^C]-labeled lysine. SIRT5 crWT and crKO cells were incubated in glutarylation medium containing radiolabeled and unlabeled lysine (EBSS, +5 mM glucose, + 50 mM Hepes, 4 μCi/ml U-[^14^C]-lysine, 0.8 mM ^12^C-lysine) for a period of 3 h, followed by quantification of released [^14^C]-CO_2_. These measurements showed that SIRT5 crKO cells had significantly reduced oxidation of lysine (by 40%) as compared to the SIRT5 crWT cells (Fig. 4*B*). These data support the notion that hyperacylation of GCDH can functionally impair flux through this pathway.

Since lysine oxidation has two discrete steps that generate CO_2_ (Fig. 4*A*), either at the dehydrogenase complex described above or at GCDH, we wanted to determine if impaired lysine oxidation observed in SIRT5 crKO cells results from reduced GCDH activity directly. For this, we modified the above-described lysine oxidation assay in a way that allowed us to directly quantify the ability of GCDH to convert glutaryl-CoA into crotonyl-CoA and CO_2_. Since glutaryl-CoA cannot enter living cells, we isolated mitochondria from the SIRT5 crWT and crKO cells that were treated with glutarylation media, in a manner similar to the lysine oxidation assay above. Mitochondria were permeabilized and incubated with [1, 5-^14^C]-glutaryl-CoA in presence of FAD, the cofactor essential for GCDH activity. After 1 h of incubation, we found that SIRT5 crKO cell mitochondria had significantly reduced ability to release radiolabeled CO_2_ (∼25%; Fig. 4*C*). We further confirmed these observations *ex vivo*, by using permeabilized liver mitochondria from 24 h fasted SIRT5WT and SIRT5KO mice and observed a similar reduction in GCDH activity (∼25%; Fig. 4*D*); GCDHKO mouse liver mitochondria served as a negative control for this assay (data not shown).

Overall, these data suggest that impaired GCDH activity could contribute to the reduced ability of SIRT5KO mice or cells to metabolize lysine, and more specifically, glutaryl-CoA. Additionally, although SIRT5 can target both glutaryl-lysine and succinyl-lysine on GCDH, SIRT5 has a greater impact on deglutarylation of GCDH and can modulate its function.

### Amino acid levels are increased in the absence of SIRT5

**Figure 5:**
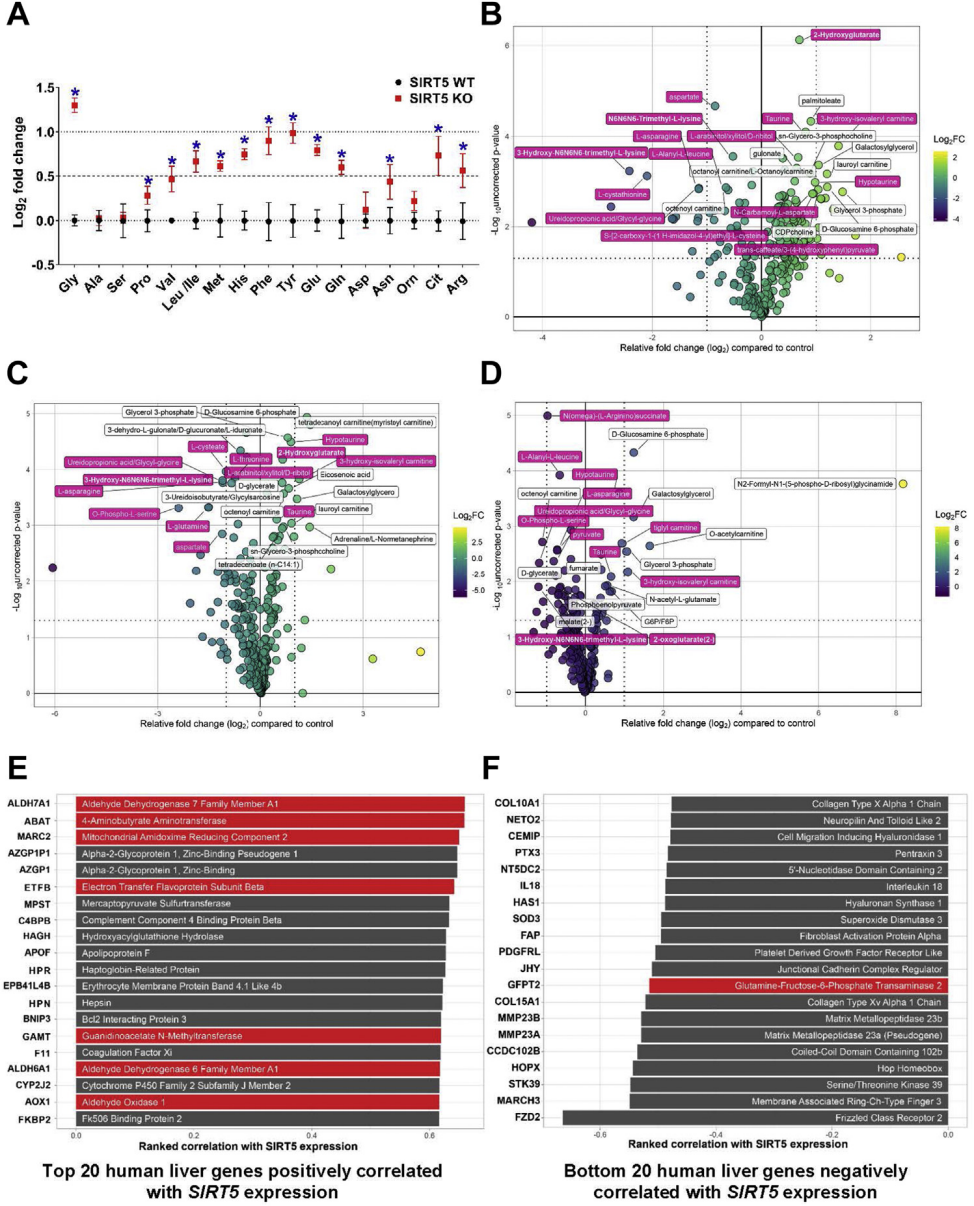
**SIRT5 expression linked to cellular amino acid levels and expression of amino acid metabolizing proteins.***A*, targeted metabolomics in 293T SIRT5 CRISPR cells reveal increased levels in the majority of amino acids in crKO compared to crWT. Data represent mean ± SD (n = 3, ∗*p*-value ≤ 0.05) of individual amino acid levels expressed as log_2_ fold change of 293T SIRT5 crKO compared to SIRT5 crWT cells. *B*–*D*, nontargeted metabolomics in 293T SIRT5 crWT and crKO cells that have been fed (*B*), fasted (*C*), or refed (*D*). Plots showing relative log2 fold change of 293T SIRT5 crKO compared to crWT reveal several amino acid metabolic intermediates (*pink*), which are among the metabolites with the largest change between genotypes with corrected *p*-value (q-value) < 0.015. *Dotted line* at 1.33 on x-axis corresponds to signficant *p*-value cutoff, while *lines* at 1 and −1 indicate a biologically significant fold change. *E* and *F*, human liver *SIRT5* coexpression analysis. Gene expressions that positively (*E*) or negatively (*F*) correlate to *SIRT5* gene expression. Genes highlighted in *red* have known roles in amino acid metabolism. SIRT5, sirtuin 5.

Because our previous results showed a decrease in lysine oxidation capacity in the absence of SIRT5, we asked whether the metabolism of other amino acids was also altered in the absence of SIRT5. To test this, we performed both targeted and nontargeted metabolomics. When we measured total levels of amino acids in SIRT5 crWT and crKO cells, we found that most amino acids exhibited increased levels in the absence of SIRT5 (Fig. 5*A*), suggesting less consumption (oxidation or protein synthesis). We hypothesized that levels of lysine intermediates, along with those of other elevated amino acids, would be altered by SIRT5 ablation. To measure lysine and its intermediates, we performed nontargeted metabolomic analyses on SIRT5 crWT and crKO cells in the fed (complete DMEM), fasted (1 h EBSS only), and refed (fasted 1 h EBSS, refed 1 h complete DMEM) states. We found significant changes in some lysine catabolic intermediates in SIRT5 crKO fed and fasted conditions as compared to the SIRT5 crWT cells (Fig. 5, *B*–*D*, *pink*; raw data Table S3). Importantly, these changes were not limited to lysine metabolism; we also observed significant changes in other amino acid metabolic intermediates (Fig. 5, *B*–*D*, *pink*). These data suggest the SIRT5 crKO phenotype goes beyond lysine catabolism, implying a broader role for SIRT5 in controlling amino acid metabolism.

### Hepatic SIRT5 expression correlates with amino acid metabolic genes

To further test the role of SIRT5 in amino acid metabolism, we performed gene expression correlation analysis using a human liver gene set. We identified a publicly available human liver gene set (NCBI GEO id 14520) measuring possible biomarkers for hepatocellular carcinoma. We filtered this dataset to only include nontumor data and performed a gene correlation analysis. We then searched for genes with the highest correlation to *SIRT5*. We found enrichment of genes involved in amino acid metabolism that positively correlated to *SIRT5* gene expression (Fig. 5, *E* and *F*, *red bars*), meaning when *SIRT5* expression is high in the human liver, expression of these genes is also high. When we looked at genes with a negative correlation to *SIRT5* expression, meaning when *SIRT5* expression is high, these genes are low, and we did not find enrichment of any processes. Furthermore, we performed these correlation analyses for the other mitochondrial sirtuins, *SIRT3* and *SIRT4*, and did not find enrichment for amino acid metabolic processes, supporting a specific role for SIRT5 in amino acid metabolism (Fig. S4, *A*–*D*).

## Discussion

SIRT5 is a mitochondrial and cytoplasmic NAD^+^-dependent protein deacylase that functions to remove a wide range of protein modifications, including malonyl-lysine, succinyl-lysine, and glutaryl-lysine. While SIRT5 has been shown to deglutarylate CPS1, little is known about protein glutarylation and how SIRT5 functions to regulate this recently discovered modification. In this study, we found the glutaryl-CoA handling enzyme, GCDH, is glutarylated, and glutarylation increases under conditions promoting lysine/tryptophan metabolism (Fig. 2). Furthermore, we demonstrated glutarylation of GCDH decreases enzymatic activity, which is partially relieved by de-glutarylation *via* SIRT5 (Fig. 3). These observations suggest a regulatory feedback loop within the lysine/tryptophan degradation pathways (Fig. 4), in which increased flux through the pathway results in the deactivation of these pathways *via* glutarylation of GCDH. Conversely, this inhibition of GCDH is relieved *via* deacylation by SIRT5.

Beyond identifying a novel substrate for SIRT5, we found a role for SIRT5 in controlling lysine metabolism. Additionally, these data indicate a broader role for SIRT5 in amino acid metabolism beyond lysine/tryptophan (Fig. 5). When integrated with previous findings of known SIRT5 targets, we propose that SIRT5 could play a broader role in regulating feedback loops within amino acid metabolic pathways. For example, catabolism of branched-chain amino acids results in increased succinyl-CoA production, which in turn could increase protein succinylation. It is possible the enzymes within the amino acid catabolic pathways are among those succinylated proteins, and modification may impact their enzymatic functions. SIRT5 is a potent desuccinylase and could abrogate protein succinylation resulting from amino acid catabolism.

We previously explored the mechanism(s) underlying this noncatalytic nature of protein acylation and reported that a specific class of 4-5 carbon dicarboxylic acyl-CoA’s possess unusual reactivity compared to monocarboxylic or shorter/longer dicarboxylic acyl-CoA species (4). We found that this enhanced reactivity results from an intrinsic property of these 4-5 carbon dicarboxylic acyl-CoA’s to undergo intramolecular catalysis and form highly reactive anhydride intermediates. The pattern of protein acylation suggested that proteins/enzymes in pathways that generate the reactive acyl-CoA species become uniquely acylated by them. While it remains unknown whether enzymes that directly handle these acyl-CoA species undergo self-acylation and if this, in turn, regulates the function of the enzyme/pathway involved, the findings presented in this study support this model. Furthermore, the concept that proteins in the vicinity of acyl-CoA’s are susceptible to nonenzymatic acylation of lysine residues was first shown by methylcrotonoyl-CoA carboxylase, an enzyme in the leucine oxidation pathway, becoming acylated by multiple acyl-CoA’s including 3-hydroxy-3-methylglutaryl-CoA, 3-methylglutaconyl-CoA, and 3-methylglutaryl-CoA, all generated within leucine oxidation or ketogenesis pathways (17).

While we are far from a complete understanding of SIRT5, how RACS behave in biological systems, and the interaction between the two, the present study provides exciting insights into this unexpected regulatory mechanism in the mitochondria. Interestingly, the role of SIRT5 in the cytoplasm or in the regulation of predominantly cytoplasmic modifications like malonylation remains enigmatic. Many studies have demonstrated that a pool of SIRT5 is found in the cytoplasm; however, we do not yet know the function of cytoplasmic SIRT5. Moreover, we do not know whether the stoichiometry of these acyl-CoA’s and the resulting modifications are enough to cause meaningful functional consequences. Additional studies will be needed to further understand the importance of SIRT5’s deacylating activity and to understand the widespread impact this system has on nutrient metabolism and cellular homeostasis.

## Experimental procedures

### Animals

All experiments in this study were reviewed and approved by the Duke University & Duke University Medical Center Institutional Animal Care and Use Committee, under Protocol Registry Number A065-20-03 which is valid until March 20, 2023.

Two to four animals per cage were housed together with a 12 h light–dark cycle and received ad libitum PicoLab rodent diet 20 (#5053, LabDiet) and water. All animal procedures were performed in accordance with the international guidelines from the Association for the Assessment and Accreditation of Laboratory Animal Care and approved by the Duke University Institutional Animal Care and Use Committee.

The SIRT5KO mice (B6;129-Sirt5tm1Fwa/J, stock #012757) were obtained from Jackson Laboratory and were backcrossed for 10 generations with C57BL/6J mice (stock #000664, Jackson Laboratory). Later, the resulting mice were backcrossed with the WT C57/BL6NJ (stock # 005304) strain from the Jackson Laboratory to obtain mice with a pure WT *Nnt* background. The resulting mice heterozygous for *Sirt5* but homozygous for WT *Nnt* were used as breeders to generate the SIRT5WT and SIRT5KO mice used in this study. For genotyping primers *Sirt5* forward (5′-AGG AGG TGG CAA AGG TCT TGC-3′), *Sirt5*KO forward (5′-TCA TTC TCA GTA TTG TTT TGC C-3′), and a common *Sirt5*WT/KO reverse (5′-CTG AGG TAG AGT CTC TCA TTG-3′) amplifying a 575 bp WT *Sirt5* band and/or a 350 bp KO *Sirt5* band were used to identify WT and SIRT5KO genotypes.

The GCDHKO mouse strain (B6.129S4-Gcdhtm1Dmk/Mmnc, # 034368-UNC) was obtained from the Mutant Mouse Regional Resource Center (a National Institutes of Health-funded strain repository #8U42OD010924-13) that was donated to the Mutant Mouse Regional Resource Center by David M. Koeller, MD, Oregon Health & Science University. These GCDHKO mice had a mixed C57/BL6J and C57/BL6NJ background, and breeders heterozygous for the *Gcdh* gene were generated by backcrossing the GCDHKO mice with the WT C57/BL6NJ strain (stock # 005304, Jackson Laboratory). By sequential genotyping and selection, mice homozygous for WT *Nnt* and heterozygous for *Gcdh* were bred to obtain the WT and GCDHKO mice used in this study. For genotyping, primer pairs *Gcdh*WT forward (5′-CTT CCG TAA CTA CTG CCA GGA GCG G-3′) and *Gcdh*WT reverse (5′-AGC TCT CGG GTC AGG AGC CCA TAG G-3′) and GcdhKO forward (5′-TTA GGC CTA GTG TGC TGG TCC CGG A-3′) and GCDHKO reverse (5′-TCT GGT GCC GGA AAC CAG GCA AAG C-3′) were used to detect either a 565 bp WT *Gcdh* band and/or a 390 bp KO band.

### Cell culture

HEK293T were obtained from Dr Eric Verdin (Gladstone Institute) and maintained at 37 °C and 5% CO_2_ in complete medium: DMEM (Gibco #11965118) supplemented with 10% (vol/vol) FBS (Thermo Fisher Scientific #26140079). These cells were used to generate the HEK293T crSIRT5KO cell line with Dharmacon’s Edit-R gene engineering system (now part of Horizon Discovery) that uses plasmid-driven Cas9 nuclease expression, synthetic trans-activating CRISPR RNA (tracrRNA), and CRISPR RNA (crRNA) for the gene of interest. As per manufacturer’s protocol, HEK293T cells at passage 17 were plated in 6-well plates at a density of 300,000 cells per well and were cotransfected with Edit-R hCMV-Puro-Cas9 (Dharmacon #U-005100-120), Edit-R tracrRNA (Dharmacon #U-002000-120), and Edit-R crRNA’s for SIRT5 using the DharmaFECT Duo (Dharmacon #T-2010-03) transfection reagent prepared in serum-free DMEM. After 48 h of transfection, cells were transferred to new 6-well plates and incubated in complete media containing 2 μg/ml puromycin for 3 days to positively select for transfected cells. Single-cell monoclonal populations were generated from puromycin-selected cells using the 96-well serial dilution technique. Guide crRNA sequences (Table S1) targeting all known transcripts of SIRT5 (NM_012241_Exon5 310 aa; NM_031244_Exon5, 299 aa; NM_001193267_Exon5, 292 aa; and NM_001242827_Exon4, 202 aa) in the sense/antisense strands of human SIRT5 were selected from Dharmacon’s (now part of Horizon Discovery) online CRISPR RNA configurator tool. Control cells underwent the same procedure as the SIRT5KO cells, except they did not receive the gene-specific crRNA. Each clonal population was cultured and screened for loss of SIRT5 protein by Western Blot. Ultimately, the confirmed SIRT5 crKO cell line was made using the crRNA ID ‘E’ (sequence in Table S1).

### Transfection and culture medium treatments

Plasmid DNA pcDNA3.1+ vector was obtained from Invitrogen (#V79020), pSG5 vector was from Agilent (#216201), WT-hSIRT5-HA was from Dr Eric Verdin (Gladstone Institute), and hGCDH-FLAG was cloned in-house in the pSG5 vector (as described below). GCDH-FLAG was overexpressed in HEK293T cells or the SIRT5 crWT/crKO cells over a period of 4 days (2 days of cell seeding followed by transfection and overexpression for 2 days). Cells are seeded in 100 mm dishes at 3 × 10ˆ5 to 5 × 10ˆ5 cells per dish and allowed to grow in the complete medium for a period of 2 days. On day 2, cells growing in complete medium were transfected with a 1:1 mixture of plasmid DNA (7.5–15 μg) and Lipofectamine 2000 reagent (Invitrogen # 11668019); both prepared in OPTI-MEM (Gibco # 31985070) as per manufacturer’s recommendations. Plasmid DNA was allowed to overexpress for additional 2 days without any additional media change. For starvation conditions, cells on day 4 were washed 1x in PBS and either complete medium (control) or acylation medium (DMEM, no glucose, no glutamine, no pyruvate, no phenol red, no serum: Gibco # A1443001), or glutarylation media (EBSS containing 5 mM glucose: Gibco # 24010043, +50 mM Hepes, +0.8 mM lysine) was added. After 3 h incubation with complete, acylation, or glutarylation media, cells were either used for metabolic assays or washed 1× in PBS and collected in immunoprecipitation (IP) lysis buffer for immunoprecipitation experiments.

### Cloning

Human *GCDH* cDNA (clone ID 3142978) in pOTB7 vector was obtained from Thermo Fisher Scientific Open Biosystems repository (now part of Horizon Discovery). To clone into pSG5 vector, an EcoRI (enzyme, NEB # 0101S) site was introduced at the 5′ end using a forward primer: 5′-CAA AAA GAA TTC ATG GCC CTG AGA GGC GTC TC-3′. At the 3′ end, the original STOP codon was removed, and a FLAG sequence followed by a new STOP codon was introduced. A BglII (enzyme, NEB # R0144S) restriction site was added at the 3′ end for cloning it into the pSG5 vector using a reverse primer: 5′-CAA AAA GAT CTT TAC TTG TCG TCA TCG TCT TTG TAG TCC TTG CTG GCC GTG AAC GCC TGG-3′. The PCR product was confirmed by EcoRI-BglII double restriction digestion and sequence-verified using Sanger sequencing.

### Immunopurification

Cells or pulverized liver tissue from 3-month-old mice were lysed in IP lysis buffer (20 mM Tris base, 150 mM sodium chloride, 1 mM EDTA, 1 mM EGTA, and 1% Triton-X 100) containing 10 mM nicotinamide, 10 mM sodium butyrate, 10 μM trichostatin A (each 10 ml containing 100 μl each of phosphatase inhibitor cocktail 2 [Millipore-Sigma # P5726] and 3 [Millipore-Sigma # P0044]), and 100 μl protease inhibitor cocktail (Millipore-Sigma # P8340), and the cell membranes were disrupted using a Series 60 Sonic Dismembrator (Model F60, Fisher Scientific) with 10 × 2 s intermittent pulses. Soluble proteins were separated from insoluble cell debris by vortex mixing and centrifugation for 1 h at 4 °C and 14,000*g*. For overexpressed proteins, immunoprecipitation was performed using anti-FLAG-M2 resin (Millipore-Sigma # A2220) and 1 to 8 mg total soluble protein per IP reaction. Briefly, the resin was washed 3× in 1× TBS (tris-buffered saline) followed by 1× wash and resuspension in lysis buffer. Soluble proteins in a total volume of 500 to 700 μl were incubated with prewashed resin in IP spin columns (Pierce cat# 69725) overnight with constant rotation at 4 °C. Next day, the spin columns were spun at 10,000*g* for 2 min, and the resin was further washed with IP lysis buffer 4× by inverting the tube for 10 times/wash. Resin-bound protein(s) were eluted by incubating samples with 50 to 100 μl of 2× Lane Marker nonreducing sample buffer (Thermo Fisher Scientific # 39001) at 95 °C for 5 min with open caps. Eluate was collected by spinning at 10,000*g* for 2 min, mixed with 2-mercaptoethanol (7.4% final concentration, Millipore-Sigma # M7522), and samples were reheated at 95 °C for 5 min. Samples were run on 10% Tris-glycine SDS-PAGE gels (BIO-RAD #4561034) for maximum separation of proteins between 50 and 25 kDa. For immunoprecipitation of endogenous SIRT5 (Millipore-Sigma #HPA022002), GCDH (Millipore-Sigma # HPA048492; Thermo # PA5-60294), glutaryl-lysine- (Cell Signaling Technologies, non-commercial #14943MF; PTMScan #26101), and succinyl-lysine- (Cell Signaling Technologies, noncommercial #13599) proteins, 1 to 10 mg of precleared lysate/500 μl of lysis buffer was incubated with 2 to 10 μg of IP antibody overnight at 4 °C. About 20 μl of protein A/G beads (Thermo Fisher Scientific # 20423) were incubated with the antigen–antibody complex at room temperature for 4 h. Normal IgG was used as a negative control in parallel with the IP reaction. For detection of endogenous immunopurified proteins, the primary antibody type (rabbit) was switched to mouse by using a conformation-specific mouse anti-rabbit antibody (Cell Signaling Technologies #3678) that only recognizes nondenatured antibodies, or light chain–specific secondary antibodies conjugated to fluorophore were used to reduce the background from the heavy and/or light chains of the denatured IP antibody. The bands were then visualized by incubating with IRDye 800CW donkey anti-mouse IgG secondary antibody (LI-COR # 926-32212) or Mouse anti-rabbit Alexa 790 light chain specific antibody (Jackson Immuno Research Labs # 211-652-171).

### Immunoblotting

Equal amounts of denatured protein samples were uniformly loaded and run on BioRad MiniPROTEAN or Criterion TGX Precast Midi Protein Gels, at 100 to 180 V for 45 to 60 min. Proteins were wet transferred to a 0.45-μm nitrocellulose or Immobilon-P (Thermo Fisher Scientific, #88520) membranes in the Bio-Rad MiniPROTEAN or Criterion Blotter. Membranes were blocked for 60 min in LI-COR blocking buffer (1× TBS, 0.45% fish gelatin, 0.1% casein, and 0.02% azide). Primary antibodies (1:1000–1:4000) were diluted in LI-COR blocking buffer or 5% BSA containing 0.1 to 0.2% Tween-20 overnight at 4 °C. Next day, membranes were washed four to six times for 5 to 10 min each in 0.1% Tween-20 containing TBS. Infrared dye-conjugated secondary antibodies were diluted 1: 10,000 in LI-COR blocking buffer containing 0.1 to 0.2% Tween-20 and incubated for 1 h at room temperature. Western blots were visualized on an Odyssey CLx imager (LI-COR). Unstripped blots were reprobed for loading control, and image analysis for quantifying band intensities was performed using the LI-COR Image Studio software (version 3.1). Antibodies: anti-MG-lysine (Millipore-Sigma # ABS2120-25UG), anti-SIRT5 (Millipore-Sigma #HPA022002 [for human SIRT5] and Cell Signaling Technologies #8782 [for mouse SIRT5]), anti-GCDH (Millipore-Sigma # HPA048492), mouse anti-β-actin (Cell Signaling Technologies, #3700), rabbit anti-β-actin (Cell Signaling Technologies #8457), anti-glutaryl-lysine (Cell Signaling Technologies, noncommercial #14943MF and PTM-Biolabs #1151), anti-succinyl-lysine (Cell Signaling Technologies, noncommercial # 13599 and PTM-Biolabs #401), anti-malonyl-lysine (Cell Signaling Technologies #14942), anti-acetyl-lysine (Cell Signaling Technologies, #9441), IRDye 680RD donkey anti-mouse IgG (LI-COR #926-68072), and IRDye 800CW donkey anti-rabbit IgG (LI-COR #926-32213).

### Quantitative polymerase chain reaction

Total RNA was isolated from cells using the Qiagen’s RNeasy kit (#74104) according to the manufacturer’s instructions (Qiagen). Total RNA (1 ug) was converted to cDNA using the iScript cDNA synthesis kit (#1708891, BIO-RAD) following the manufacturer’s protocol (BIO-RAD). Quantitative real-time PCR was performed on an Applied Biosciences ViiA 8 thermocycler (Thermo Fisher Scientific) using iTaq Universal SYBR Green Supermix (#1725121, BIO-RAD) and the following primer sets: SIRT3-Forward (5′-GCT GTA CCC TGG AAA CTA CAA-3′), SIRT3-Reverse (5′-ATC GAT GTT CTG CGT GTA GAG-3′), SIRT4-Forward (5′-GAG CTT TGC GTT GAC TTT CAG-3′), SIRT4-Reverse (5′-GGA CTT GCT GGC ACA AAT AAC-3′), SIRT5-Forward (5′-GCT CGG CCA AGT TCA AGT AT-3′), SIRT5-Reverse (5′-AAG GTC GGA ACA CCA CTT TC-3′), ATPSB-Forward (5′-CTA GAC TCC ACC TCT CGT ATC A-3′), and ATPSB-Reverse (5′-CAT ACC CAG GAT GGC AAT GA-3′). Relative amounts of *SIRT3*, *SIRT4*, and *SIRT5* mRNA were calculated using the ΔΔCt method with ATP synthase subunit B as the endogenous control. Statistical comparisons were made using multiple *t* test with Benjamini–Hochberg correction for multiple comparisons.

### Lysine oxidation assay

SIRT5 crWT and SIRT5 crKO cells were plated at 1∗10ˆ5 cells/well in 12-well plates in a complete medium (DMEM/10% FBS). After 24 h, cells were washed 1× in PBS, and 1 ml assay media containing 4 μCi/ml U-[^14^C]-lysine (Moravek Inc #MC197 250UCI), 0.8 mM ^12^C-lysine, 50 mM Hepes, pH 7.5 in EBSS were added. Cells were incubated in this medium for 3 h after which 750 μl of the assay medium was aspirated and transferred to 13-mm culture tubes that contained a 200 μl 1N NaOH trap. The [^14^C]-CO_2_ generated as a result of lysine oxidation was released from the bicarbonate buffer by acidifying the medium with 70% perchloric acid. Tubes were incubated on a shaking water bath for 1 h at 37 °C. After 1 h, NaOH from the trap was carefully removed and added directly into scintillation vials containing the Uniscint BD scintillation fluid (National Diagnostics #LS-276). Disintegrations per minute were counted on an LS 6500 scintillation counter (Beckman Coulter). The amount of label released was background subtracted and expressed as a percentage of total radiolabel added in the incubation medium. Statistical analysis was performed using a two-tailed unpaired *t* test to compare SIRT crWT *versus* SIRT crKO cells.

### Isolation of enriched mitochondrial fraction

Fresh mouse liver or cells were rinsed in ice-cold PBS and homogenized in 10 volumes of STE buffer (0.25 M sucrose, 10 mM Tris-HCl, pH 8.0, 1 mM EDTA, and 1× HALT protease inhibitor cocktail; Thermo Fisher Scientific #78420B) with 15 strokes in a chilled glass-Teflon homogenizer. The homogenate was centrifuged at 700*g* for 10 min at 4 °C, and the resulting supernatant was centrifuged at 7000*g* for 10 min at 4 °C. The pellet from the 7000*g* centrifugation was used as the mitochondrial enriched fraction for glutaryl-CoA oxidation assay and BN-PAGE.

### Glutaryl-CoA oxidation assay

Mitochondria were isolated from fresh livers of male SIRT5WT and SIRT5KO mice (3–4 months old with GCDHKO mouse liver mitochondria as negative control) or cells as described above. Mitochondria were permeabilized by resuspending the mitochondrial pellet in permeabilization buffer (105 mM K-MES, pH 7.1, 30 mM KCl, 10 mM KH_2_PO_4_, 5 mM MgCl_2_, 0.5 mg/ml BSA, and 30 μg/ml alamethicin; Sigma-Aldrich #A5362) at a protein concentration of 3 to 5 μg/μl and incubated for 5 min on ice. GCDH activity assay was initiated by mixing 100 ul of permeabilized mitochondria with 100 μl of GCDH reaction buffer (105 mM K-MES, pH 7.1, 30 mM KCl, 10 mM KH_2_PO_4_, 5 mM MgCl_2_, 0.5 mg/ml BSA, 30 μg/ml alamethicin, 200 μM FAD, and 0.1 μCi/μl [1, 5-^14^C]-glutaryl-CoA; American Radiolabeled Chemicals Inc. #ARC3471). The reaction was incubated for 30 min at 37 °C, and the [^14^C]-CO_2_ produced was trapped and quantified as described for the lysine oxidation assay. Statistical analysis was performed using a two-tailed unpaired *t* test to compare SIRTWT *versus* SIRTKO mouse liver lysates.

### Blue-native polyacrylamide gel electrophoresis

Liver mitochondria were isolated from 24 h fasted male SIRT5WT and SIRT5KO mice (2 month old, age-matched GCDHWT and GCDHKO mice were used as controls) using the above-mentioned procedure and stored at −80 °C. Thawed mitochondrial pellets were solubilized in 1× Native-PAGE sample buffer containing 2% n-dodecyl-β-D-maltoside, 0.5% G-250, and 1× inhibitors (protease inhibitors, phosphatase inhibitors, nicotinamide, trichostatin A, and sodium butyrate) as per manufacturer guidelines for NativePAGE Sample Prep Kit (Thermo Fischer Scientific #BN2008). Equal amounts of protein (15 μg) were loaded on NativePAGE Novex 4 to 16% Bis-Tris Gels and were run at 150 V for the first 60 min followed by another 60 min at 250 V. Proteins were transferred to PVDF membranes by wet transfer method mentioned above. Posttransfer PVDF membranes were washed 3× in 100% methanol for 5 min each to remove excess G250 dye and were processed for immunoblotting as mentioned above. Statistical analysis was performed using a two-tailed unpaired *t* test to compare SIRTWT *versus* SIRTKO mouse liver lysates.

### Chemical acylation and deacylation of recombinant human GCDH protein

Recombinant human GCDH was expressed and purified to homogeneity as described in (18). Glutaryl-GCDH (or succinyl-GCDH) was prepared by incubating the protein with excess glutaryl-CoA (or succinyl-CoA). Briefly, 20 μM GCDH was incubated with 1.6 mM glutaryl-CoA/succinyl-CoA, in acylation buffer (50 mM Hepes pH 8.0 and 150 mM NaCl) for 15 h at 25 °C, with gentle stirring. After incubation, the reaction mixture was applied to a Superdex 200 Increase 10/300 GL gel filtration column (GE Healthcare Life Sciences #28-9909-44) to remove excess glutaryl-CoA/succinyl-CoA. Modified GCDH was eluted at a rate of 0.75 ml/min with elution buffer (25 mM Hepes pH 7.8 and 30 mM NaCl). The Superdex 200 column was previously calibrated using standard proteins (Ferritin, ovalbumin, ribonuclease A, conalbumin, and carbonic anhydrase) which were used to determine the molecular mass of the pure modified proteins. The tetrameric form of the pure unmodified/modified proteins was used in subsequent structural and enzymatic activity assays. For diacylation, recombinant 22.5 μg SIRT5 (9) was incubated with glutaryl-GCDH or succinyl-GCDH (135 μg) in 5 mM Tris-HCl pH 9, 4 mM MgCl_2_, 5 mM NaCl, 0.5 mM DTT, and 1 mM NAD^+^, for 3 h at 37 °C. Unmodified GCDH and modified GCDH with or without SIRT5 were used as controls for this deacylation reaction.

### GCDH activity assay

GCDH activity was evaluated using two distinct enzymatic assays. In the first assay, GCDH activity was measured at 25 °C, monitoring the reduction of 2,6-dichlorophenolindophenol (DCPIP, 30 μM, Millipore-Sigma #36180) at 600 nm, upon energization with the substrate glutaryl-CoA (15 μM) in the presence of the PMS (2 mM, Millipore-Sigma #P9625) mediator, as in (18). In the second assay, the activity of GCDH was inferred from its ability to reduce the ETF, which is its physiological electron acceptor. For this, GCDH was incubated with glutaryl-CoA (15 μM) and purified recombinant human ETF (19, 20) and the ability of ETF to receive electrons from GCDH was monitored by measuring DCPIP reduction at 600 nm (21). Buffer for enzymatic assays was 10 mM Hepes pH 7.8. One unit of catalytic activity is defined as nmol of DCPIP reduced per minute, under conditions used in the assay. The activity of glutarylated GCDH and deglutarylated GCDH was calculated as a percentage of unmodified GCDH controls. All measurements were made in quadruplicates, and statistical analysis was performed using a one-way ANOVA with Tukey’s post hoc test to make multiple comparisons between unmodified, modified, and de-acylated (SIRT5 co-incubation) GCDH samples.

### Spectroscopic methods

Fluorescence spectroscopy was performed using a Jasco FP-8200 spectrofluorometer with a cell holder thermostatically controlled with a Peltier. For tryptophan emission, the excitation wavelength was set at 280 nm, and FAD emission was followed by setting excitation wavelength at 450 nm; slits were 5 and 10 nm for excitation and emission, respectively. Typically, protein concentration was 2 μM. CD spectra were recorded for GCDH on a Jasco J-1500 spectropolarimeter with a cell holder thermostatically controlled with a Peltier. A quartz polarized 1 mm path length quartz cuvette (Hellma) was used, and protein concentrations were typically 0.1 mg/ml.

### Thermal stability

Thermal unfolding with a linear temperature increase was followed using CD (ellipticity variation at 222 nm) and fluorescence spectroscopy (tryptophan emission λ_ex_ =280 nm and λ_em_ =340 nm and FAD emission λ_ex_ =450 nm and λ_em_ =530 nm). In all experiments, a heating rate of one °C/min was used, and temperature was increased from 20 to 85 °C. Data were analyzed according to a two-state model, and fits to the transition curves were made using OriginPro8.

### Label-free qualitative acyl-proteomic analysis of recombinant GCDH

Chemically glutarylated and succinylated human recombinant GCDH protein was prepared as mentioned above. About 10 to 30 ug protein was loaded on 10% NuPAGE Bis-Tris protein gels (Thermo Fischer Scientific #NP0301BOX) and separated using the NuPAGE MOPS SDS running buffer (Thermo Fischer Scientific #NP0001) for 1 h at 180 V. Postrun, the gel was stained overnight in colloidal blue stain (Thermo Fischer Scientific #LC6025), washed in de-ionized water, and bands corresponding to ∼44 kDa were excised and collected in 1.5 ml microcentrifuge tubes. Gel pieces were cut into smaller pieces using a sterile 20 μl pipette tip, vortex mixed, and incubated twice in 200 μl of a 1:1 mixture of acetonitrile (ACN) and 100 mM ammonium bicarbonate (AmBIC) at room temperature for 15 min followed by dehydration in 200 μl of 100% ACN from 1 min. The gel pieces were rehydrated and reduced with 200 μl 10 mM dithiothreitol (DTT) in 100 mM AmBIC for 30 min at 55 °C. After discarding the DTT solution, the samples were alkylated using 200 μl of 55 mM iodoacetamide in 100 mM AmBIC in dark at room temperature for 30 min. Samples were further washed in 200 μl of 1:1 ACN: 100 mM AmBIC mixture followed by 200 uL of 100% ACN. Samples were rehydrated in 200 μl of digestion buffer (50 mM AmBIC, 5 mM CaCl2) containing 1 ug trypsin per 50 μg of GCDH protein and in-gel digested overnight at 37 °C. Trypsin digested peptides were serially extracted using 200 μl each of 50% ACN, 0.3% formic acid, and 80% ACN, 0.3% formic acid at room temperature for 15 min each. Pooled extracts were subjected to speed-vac until dry and resuspended in 1 ml of 0.5% trifluoroacetic acid. Acidified samples were then solid-phase desalted by solid phase extraction using 50 mg tC18 Sep-Pak columns (Waters) and dried using speed-vac.

For assessment of glutarylated and deglutarylated GCDH, samples were resuspended in 22 μl of 0.1% formic acid and subjected to nanoLC-MS/MS analysis using a using an EASY-nLC UPLC system (Thermo Fisher Scientific) coupled to a Q Exactive Plus Hybrid Quadrupole-Orbitrap mass spectrometer (Thermo Fischer Scientific) *via* a nanoelectrospray ionization source. To minimize the effects of acylpeptide carryover, we assessed unmodified GCDH first and blanks in between samples. Sample injections of 10 μl were first trapped on an Acclaim PepMap 100 C18 trapping column (3 um particle size, 75 μm × 20 mm) with 22 μl of solvent A (0.1 % FA) at a variable flow rate dictated by max pressure of 500 Bar, after which the analytical separation was performed over a 105 min gradient (flow rate of 300 nl/minute) of 5 to 40% solvent B (90% ACN, 0.1% FA) using an Acclaim PepMap RSLC C18 analytical column (2 um particle size, 75 μm × 500 mm column (Thermo Fischer Scientific) with a column temperature of 55 °C. MS^1^ (precursor ions) was performed at 70,000 resolution, with an AGC target of 3 × 10^6^ ions and a maximum injection time of 100 ms. MS^2^ spectra (product ions) were collected by data-dependent acquisition of the top 20 most abundant precursor ions with a charge greater than one per MS1 scan, with dynamic exclusion enabled for a window of 30 s. Precursor ions were filtered with a 1.2 m/*z* isolation window and fragmented with a normalized collision energy of 27. MS2 scans were performed at 17,500 resolution, with an AGC target of 1 × 10^5^ ions and a maximum injection time of 100 ms. Similar methods were used for direct comparison of PTMs resulting from glutaryl and succinyl-CoA reactions.

Raw LC-MS/MS data were processed in Proteome Discoverer v2.2 (PD2.4, Thermo Fisher Scientific), using the Sequest HT and MS Amanda 2.0 (Protein Chemistry Facility IMP/IMBA/GMI) search engines as nodes. Data were searched against the UniProt *E. coli* (strain K12) complete proteome database of reviewed (Swiss-Prot) and unreviewed (TrEMBL) proteins, which consisted of 4276 sequences on the date of download (3/15/18) and hGCDH-6XHIS (modified from UniProtKB - Q92947). Variable modifications included oxidation of methionine (M) and glutarylation (114.0316941 Da monoisotopic mass) of lysine (K), with carbamidomethyl of cysteine (C) as a fixed modification. Data were searched with a 10 ppm precursor mass and 0.02 Da product ion tolerance. The maximum number of missed cleavages was set at four and enzyme specificity was designated as trypsin. Peptide spectral matches were filtered to a 1% false discovery rate (FDR) with Percolator (22). Site localization probabilities were determined using ptmRS (23) using a 75% Site Probability Threshold. Peptide spectral matches were grouped to peptides maintaining 1% FDR at the peptide level, and peptides were grouped to proteins using the rules of strict parsimony. Proteins were filtered to 1% FDR using Protein FDR Validator. Peptide quantification was done using the MS1 precursor intensity from aligned features (10 min max retention time shift), and imputation was performed *via* low abundance resampling. Quantitation for each GCDH acylpeptide identified was normalized to the relative abundance of hGCDH-6XHIS protein (calculated from unmodified GCDH peptides only) to control for any slight deviations in sample loading and LC-MS performance. Statistical significance was assessed by taking the Log2 of GCDH-normalized acylpeptide abundance values and calculating Log_2_ fold-changes and performing the Student’s *t* test (n = 3) across conditions. The mass spectrometry proteomics data have been deposited to the ProteomeXchange Consortium *via* the PRIDE (24) partner repository with the dataset identifier PXD018156.

### Molecular modeling of GCDH-acyl sites

The crystal structure coordinates for the homotetramer of human glutaryl-CoA dehydrogenase (25) were downloaded from the Protein Data Bank (PDB ID: 1SIR; www.rcsb.org; validation report: https://files.rcsb.org/pub/pdb/validation_reports/si/1sir/1sir_full_validation.pdf). The structure was assessed to correct the protonation states, predict side chain pKa, optimize the intramolecular/intermolecular hydrogen bonding networks (26, 27) and add the two missing C-terminal residues to each subunit using YASARA Structure v17.4 (www.yasara.org). The protein structure was then typed with the CHARMM force field (28) and subjected to energy minimization with the Generalized Born with simple switching implicit-solvent model to a root mean square convergence of <0.01 kcal/mol using Biovia Discovery Studio 2018 (www.3dsbiovia.com). Figures were generated using Lightwave3D 2018 (www.lightwave3d.com).

### Targeted metabolomics

293T SIRT5 crWT and crKO cells were collected during the exponential growth phase, and metabolites were isolated and analyzed using a stable isotope dilution technique. Amino acids and acylcarnitine measurements were made by flow injection tandem mass spectrometry using sample preparation methods described previously (29, 30). The data were acquired using a Waters TQD mass spectrometer equipped with Acquity UPLC system and controlled by MassLynx 4.1 operating system (Waters). Statistical analysis was performed using multiple *t* tests to compare individual amino acids present in SIRT5 crKO *versus* crWT cell extracts (n = 3 per group).

### Nontargeted metabolomics in SIRT5 CRISPR cells

Nontargeted metabolomics were performed using Sirt5 crWT and crKO 293T cells that were either fed (complete DMEM, [Gibco #11965118] supplemented with 10% [vol/vol] FBS [Thermo Fisher Scientific #26140079]), fasted (1 h EBSS with phenol red), or refed (fasted 1 h in EBSS with phenol red and refed for 1 h in complete DMEM). Cells were prepared for metabolomics by first removing media and then placing on dry ice and adding precooled 80% methanol/water (v/v; HPLC grade). Enzymes were further inactivated by incubating plate(s) in −80 °C freezer 15 min. Cells were scraped into the solvent, transferred to tubes, and centrifuged at 20,000 rcf, 10 min at 4 °C. Samples were dried using a speed vacuum at room temperature, and pellets were stored at −80 °C until processing for LC-MS.

Metabolite extraction was performed as described in previous study (31). The supernatant was transferred to a new Eppendorf tube and dried in vacuum concentrator at room temperature. The dry pellets were reconstituted into 30 ml sample solvent (water:methanol:acetonitrile, 2:1:1, v/v), and 3 ml was further analyzed by LC-MS. Ultimate 3000 UHPLC (Dionex) is coupled to Q Exactive Plus-Mass spectrometer (Thermo Scientific) for metabolite profiling. A hydrophilic interaction chromatography method employing an Xbridge amide column (100 × 2.1 mm i.d., 3.5 mm; Waters) is used for polar metabolite separation. Detailed LC method was described previously(32) except that mobile phase A was replaced with water containing 5 mM ammonium acetate (pH 6.8). The Q Exactive Plus-Mass spectrometer is equipped with a HESI probe with related parameters set as below: heater temperature, 120 °C; sheath gas, 30; auxiliary gas, 10; sweep gas, 3; spray voltage, 3.0 kV for the positive mode, and 2.5 kV for the negative mode; capillary temperature, 320 °C; S-lens, 55; and a scan range (m/z) of 70 to 900 was used in positive mode from 1.31 to 12.5 min. For negative mode, a scan range of 70 to 900 was used from 1.31 to 6.6 min and then 100 to 1000 from 6.61 to 12.5 min; resolution: 70,000; and automated gain control, 3 × 106 ions. Customized mass calibration was performed before data acquisition. LC-MS peak extraction and integration were performed using commercially available software Sieve 2.2 (Thermo Scientific). The peak area was used to represent the relative abundance of each metabolite in different samples. The missing values were handled as described in a previous study (32). Statistical analysis was performed using a two-tailed unpaired *t* test to compare SIRT5 crWT *versus* crKO cell extracts (n = 3 per group) in R version 3.5.2 using the following packages: tidyverse, readxl, janitor, viridis, ggrepel, broom, and svglite.

### Bioinformatics

The code for gene correlation is available within the Hirschey Lab Github (https://github.com/matthewhirschey/livercoexpression).To summarize, liver gene expression values, IDs, and descriptions were downloaded from [NCBI GEO]; (https://www.ncbi.nlm.nih.gov/geo/query/acc.cgi?acc=GSE14520). Data were cleaned, tidied, and prepared for analysis within R using the `tidyverse` package. Liver samples were filtered to include only nontumor samples. A correlation matrix was built to identify similar patterns of hepatic expression for all genes compared to all genes using the `corrr` package. Probe IDs for *SIRT3*, *SIRT4*, and *SIRT5* were queried and summarized for the top 20 gene correlations. To measure the distribution of correlation coefficients, r^2^ values were resampled from the entire matrix to generate 1000 'virtual' gene lists using the package `moderndive`. Mean and standard deviations were calculated from this virtual set. A standard deviation threshold of ± two standard deviations was set, and the resulting gene correlations with each gene of interest greater than or less than the threshold were considered statistically unlikely by chance and then queried for gene set enrichment analysis.

### Statistical analyses

Unless otherwise indicated, figure data represent the mean ± standard deviation. Significance for comparison between two groups was evaluated using a two-tailed unpaired Student’s *t* test or a one-way ANOVA followed by an appropriate posthoc test (parametric or nonparametric) for comparisons between more than two groups. A result was considered significant if *p*-value ≤ 0.05 (∗). Statistical tests and graphs were prepared using GraphPad Prism 7.0 software

## Data availability

Proteomic data files have been deposited on ProteomeXchange with the accession number PXD018156. Code for computational analyses is available on Github (https://github.com/matthewhirschey/livercoexpression).

## Supporting information

This article contains supporting information.

## Conflict of interest

The authors declare that they have no conflicts of interest with the contents of this article.

## Supporting information

Supplemental Table S1 CRISPR RNA guide sequences and target chromosomal location.

Supplemental Table S2 List of acylation sites on Recombinant GCDH (Related to Fig. 3). Supplementary Excel file containing label-free quantitative mass spectrometry data assessing glutarylation of lysines on hGCDH-6xHis after chemical glutarylation and incubation with or without mSirt5. Data are shown on the following spreadsheet tabs: Tab 1) GCDH Glutaryl Site Summary: Summary of relative quantitation statistics for glutarylation at individual sites on hGCDH-6His, calculated from a subset of peptides identified at 1% FDR (Tab2). Some sites combine quantitation from multiple GCDH glutaryl-peptides, all normalized for hGCDH-6xHis abundance in each sample (calculated from unmodified GCDH peptides only). Tab 2) Peptide Groups: Output from Proteome Discoverer 2.4, showing information on all peptides identified at 1% FDR. Column J highlights peptides mapping to hGCDH-6His (yellow) or mSirt5 (orange), while column Q highlights site localization probabilities for GCDH glutaryl-peptides (green). The “Abundances (Normalized)” values (columns AO-AW) were used for summing quantification for peptides with common glutaryl-sites, applying log_2_-normalization, and testing for statistical significance (Tab1). Tab 3) Protein Groups: Output from Proteome Discoverer 2.4, showing information on all “Master Proteins”, or representatives of protein groups to which the peptides in Tab2 map at 1% protein-level FDR. Rows are colored to highlight hGCDH-6His (yellow) and mSirt5 (orange) and are rank-ordered by Coverage (Column N), with hGCDH—6His as the top protein at 100% sequence coverage. The hGCDH-6His (Row 2, yellow) “Abundances (Normalized)” values (columns BI-BQ) are equal for all samples as all quantitation was normalized to hGCDH-6His levels in each sample.

Supplemental Table S3 crSIRT5 raw metabolomics data. This table contains the raw metabolomics data associated with Figure 5. Analysis of these data is detailed in the Non-targeted metabolomics section of the materials and methods.

Supplemental Figure S1 **Validation of HEK293T CRISPR SIRT5 KO cells.***A*, relative SIRT5 protein quantification of (Fig. 1*C*) (mean ± SD, n = 3). *B*, relative mRNA expression of mitochondrial sirtuins (*SIRT3*, *4* and *5*) normalized to ATP subunit 5 mRNA levels in 293T SIRT5 crWT and crKO cells (mean ± SEM, n = 5, ∗*p*-value ≤ 0.05). *C*, immunoblot of SIRT5 crWT and crKO whole cell lysates blotted using acetyl-lysine antibody. *D*, relative quantification of acetylated proteins normalized to β-actin levels (mean ± SD, n = 3).

Supplemental Figure S2 **GCDH Succinylation.***A*, immunoblot for immuno-purified GCDH-FLAG by Flag-M2 resin or succinyl-proteins by anti-succinyl-lysine antibody (Succ-K) from 293T SIRT5 CRISPR cells grown in complete media. Blots representative of at least three independent experiments. Same experiment as Figure 2*A*, blotted for Succ-K. *B*, immunoblot for immuno-purified GCDH-FLAG by Flag-M2 resin from 293T SIRT5 crWT or crKO cells grown in complete media or acylation media (DMEM without glucose, glutamine, pyruvate, 10% FBS). Blots are representative of at least three independent experiments and quantitative values of succinyl-lysine intensity normalized to FLAG intensity expressed as ratios relative to control (overexpressed GCDH-FLAG in SIRT5 crWT cells grown in complete media, lane 2. Same experiment as Fig. 2*B*, blotted for Succ-K). *C*, immunoblot of immuno-purified GCDH-FLAG by Flag-M2 resin from 293T SIRT5 CRISPR cells grown in complete media with or without co-expressed SIRT5-HA. Blots representative of at least three independent experiments and quantitative values of succinyl-lysine intensity normalized to FLAG intensity expressed as rations relative to control (overexpressed GCDH-FLAG, lane 2. Same experiment as Fig. 2*C*, blotted for Succ-K). *D*, immunoblot for immuno-purified GCDH-FLAG by Flag-M2 resin from SIRT5 crWT or crKO cells grown in complete media or glutarylation media (EBSS containing 5 mM glucose, +50 mM HEPES, +0.8 mM Lysine) with or without co-expressed SIRT5-HA. Blots are representative of at least three independent experiments and quantitative values of glutaryl-lysine intensity normalized to FLAG intensity expressed as ratios relative to control (overexpressed GCDH-FLAG in SIRT5 crWT cells grown in complete media, lane 1. Same experiment as Fig. 2*D*, blotted for Succ-K).

Supplemental Figure S3 **SIRT5 has little impact on Succinylation of GCDH****.***A*, immunoblot of chemically modified GCDH (hGCDH-6xHIS) using succinyl-CoA with or without incubation with recombinant SIRT5 (mSIRT5-6xHIS). Quantitative values of succinyl-lysine intensity normalized to total protein are expressed as ratios relative to control (succinyl-modified recombinant GCDH, lane 2). *B*,enzymatic activity of unmodified (black), succinylated (dark green) and desuccinylated (bright green) GCDH determined by using PMS/DCPIP as artificial electron acceptors and Succinyl-CoA as electron donor (mean ± SD, n = 6, 8, and 7 for unmodified, modified and deacylated GCDH respectively; ∗*p*-value ≤ 0.05). *C*, enzymatic activity of unmodified (black), succinylated (dark green) and de-succinylated (light green) GCDH determined by using ETF/DCPIP as an electron acceptor and Succinyl-CoA as electron donor (mean ± SD, n = 4, 4, and 3 for unmodified, modified and deacylated GCDH respectively, ∗*p*-value ≤ 0.05). *D*, succinyl-K sites as identified by label-free quantitative LC-MS/MS on recombinant GCDH are mapped on full-length human GCDH protein. *E* and *F*, homo-tetrameric structure of human GCDH with each subunit colored separately (purple, blue, red, and green). Yellow sticks indicate modified lysine residues. The FAD cofactor and CoA substrate are black and light gray spheres, respectively. *G*, far-UV CD spectra for succinylated GCDH (green) and unmodified GCDH (black). *H*, thermal stability profiles for succinylated-GCDH (open circles, green curve) and unmodified GCDH (closed squares, green curve). The solid lines represent two-state sigmoid curves from which the apparent midpoint temperature was determined. *I*, immunoblot of native PAGE separated GCDH tetramer and SDS-PAGE for denatured GCDH isolated from liver mitochondria of 24 h fed WT and *Sirt5*^-/-^ (SIRT5KO) mice. Fed *Gcdh^-/-^* (GCDHKO) mouse liver mitochondrial lysates were used as a control to identify the correct GCDH tetramer complex. *J*, quantitation of GCDH tetramer intensity normalized to ETF complex intensity. Data representative of two independent experiments (mean ± SD, n = 3 for both SIRT5WT and SIRT5KO mice).

Supplemental Figure S4 **Human liver co-expression analy****sis for *SIRT3* and *4*.***A* and *B*, human liver *S**IRT**3* liver co-expression analysis showing gene expressions that positively (*A*) or negatively (*B*) correlate to *S**IRT**3* gene expression. *C* and *D*, human liver *S**IRT**4* liver co-expression analysis showing gene expressions that positively (*C*) or negatively (*D*) correlate to *S**IRT**4* gene expression. Genes highlighted in red have known roles in amino acid metabolism.
