# Supplementary figures and images for: Deglutarylation of glutaryl-CoA dehydrogenase by deacylating enzyme SIRT5 promotes lysine oxidation in mice
Source: J Biol Chem. 2022 Feb 12;298(4):101723. doi: 10.1016/j.jbc.2022.101723 (PMC8969154; doi:10.1016/j.jbc.2022.101723)

Figure S1

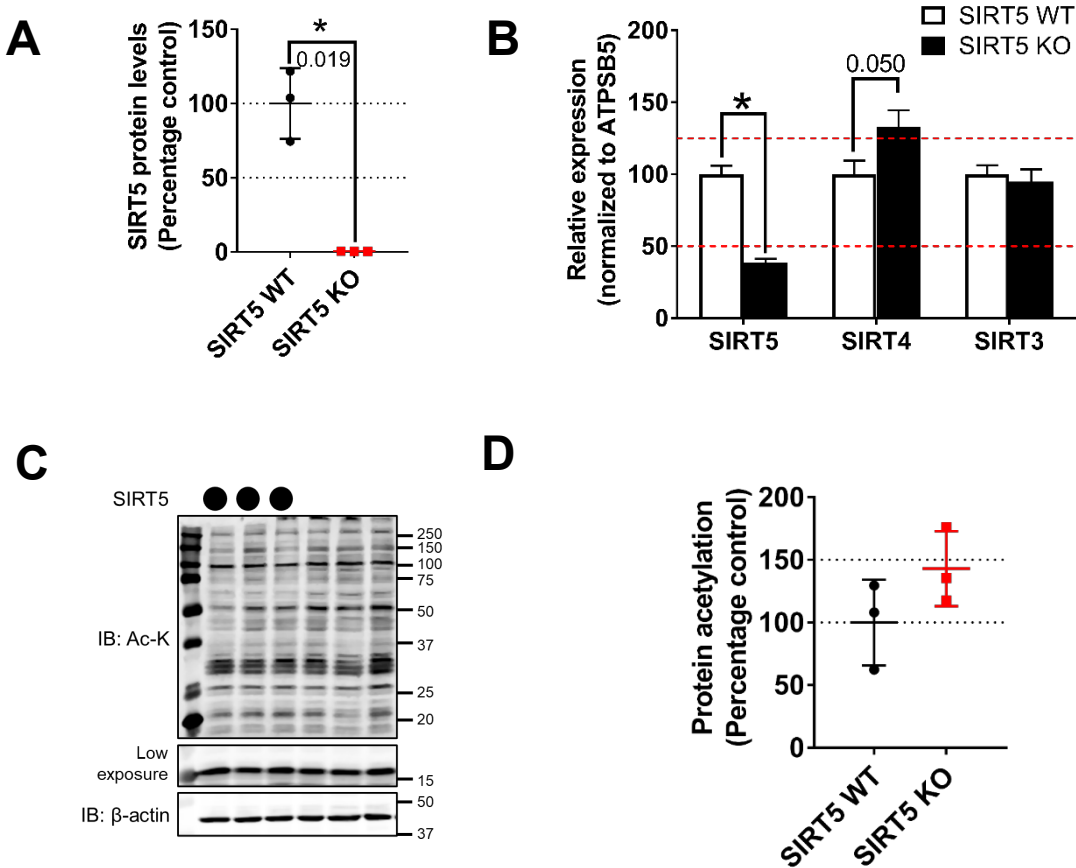

Supplement: Supplemental Figure S1 — Validation of HEK293T CRISPR SIRT5 KO cells.A, relative SIRT5 protein quantification of (Fig. 1C) (mean ± SD, n = 3). B, relative mRNA expression of mitochondrial sirtuins (SIRT3, 4 and 5) normalized to ATP subunit 5 mRNA levels in 293T SIRT5 crWT and crKO cells (mean ± SEM, n = 5, ∗p-value ≤ 0.05). C, immunoblot of SIRT5 crWT and crKO whole cell lysates blotted using acetyl-lysine antibody. D, relative quantification of acetylated proteins normalized to β-actin levels (mean ± SD, n = 3). [file mmc4.pdf]

# A

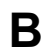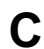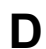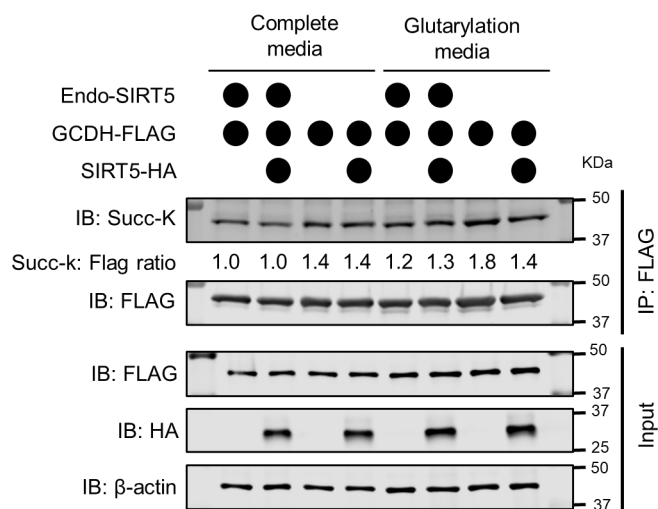

Supplement: Supplemental Figure S2 — GCDH Succinylation.A, immunoblot for immuno-purified GCDH-FLAG by Flag-M2 resin or succinyl-proteins by anti-succinyl-lysine antibody (Succ-K) from 293T SIRT5 CRISPR cells grown in complete media. Blots representative of at least three independent experiments. Same experiment as Figure 2A, blotted for Succ-K. B, immunoblot for immuno-purified GCDH-FLAG by Flag-M2 resin from 293T SIRT5 crWT or crKO cells grown in complete media or acylation media (DMEM without glucose, glutamine, pyruvate, 10% FBS). Blots are representative of at least three independent experiments and quantitative values of succinyl-lysine intensity normalized to FLAG intensity expressed as ratios relative to control (overexpressed GCDH-FLAG in SIRT5 crWT cells grown in complete media, lane 2. Same experiment as Fig. 2B, blotted for Succ-K). C, immunoblot of immuno-purified GCDH-FLAG by Flag-M2 resin from 293T SIRT5 CRISPR cells grown in complete media with or without co-expressed SIRT5-HA. Blots representative of at least three independent experiments and quantitative values of succinyl-lysine intensity normalized to FLAG intensity expressed as rations relative to control (overexpressed GCDH-FLAG, lane 2. Same experiment as Fig. 2C, blotted for Succ-K). D, immunoblot for immuno-purified GCDH-FLAG by Flag-M2 resin from SIRT5 crWT or crKO cells grown in complete media or glutarylation media (EBSS containing 5 mM glucose, +50 mM HEPES, +0.8 mM Lysine) with or without co-expressed SIRT5-HA. Blots are representative of at least three independent experiments and quantitative values of glutaryl-lysine intensity normalized to FLAG intensity expressed as ratios relative to control (overexpressed GCDH-FLAG in SIRT5 crWT cells grown in complete media, lane 1. Same experiment as Fig. 2D, blotted for Succ-K). [file mmc5.pdf]

Figure S3:

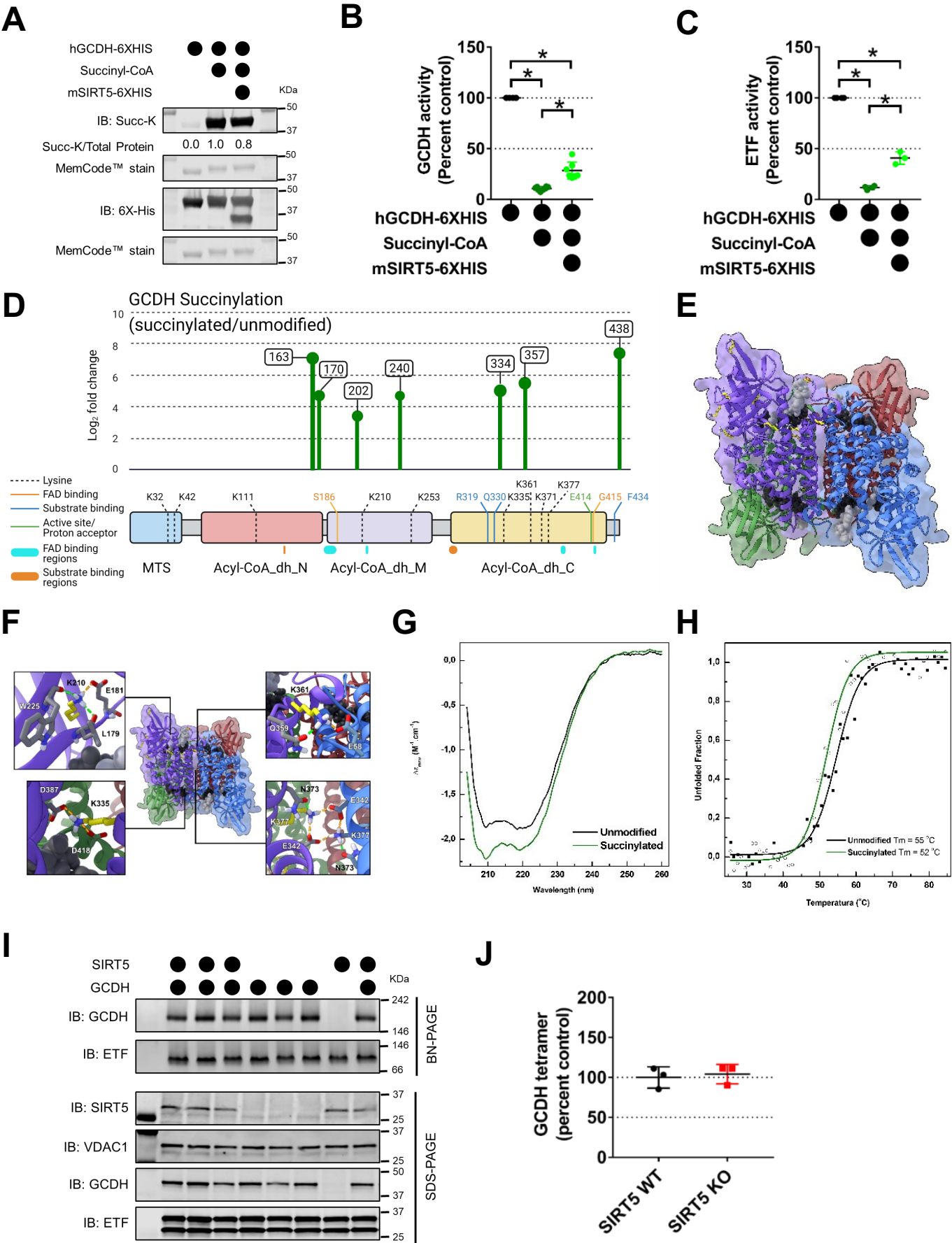

Supplement: Supplemental Figure S3 — SIRT5 has little impact on Succinylation of GCDH.A, immunoblot of chemically modified GCDH (hGCDH-6xHIS) using succinyl-CoA with or without incubation with recombinant SIRT5 (mSIRT5-6xHIS). Quantitative values of succinyl-lysine intensity normalized to total protein are expressed as ratios relative to control (succinyl-modified recombinant GCDH, lane 2). B,enzymatic activity of unmodified (black), succinylated (dark green) and desuccinylated (bright green) GCDH determined by using PMS/DCPIP as artificial electron acceptors and Succinyl-CoA as electron donor (mean ± SD, n = 6, 8, and 7 for unmodified, modified and deacylated GCDH respectively; ∗p-value ≤ 0.05). C, enzymatic activity of unmodified (black), succinylated (dark green) and de-succinylated (light green) GCDH determined by using ETF/DCPIP as an electron acceptor and Succinyl-CoA as electron donor (mean ± SD, n = 4, 4, and 3 for unmodified, modified and deacylated GCDH respectively, ∗p-value ≤ 0.05). D, succinyl-K sites as identified by label-free quantitative LC-MS/MS on recombinant GCDH are mapped on full-length human GCDH protein. E and F, homo-tetrameric structure of human GCDH with each subunit colored separately (purple, blue, red, and green). Yellow sticks indicate modified lysine residues. The FAD cofactor and CoA substrate are black and light gray spheres, respectively. G, far-UV CD spectra for succinylated GCDH (green) and unmodified GCDH (black). H, thermal stability profiles for succinylated-GCDH (open circles, green curve) and unmodified GCDH (closed squares, green curve). The solid lines represent two-state sigmoid curves from which the apparent midpoint temperature was determined. I, immunoblot of native PAGE separated GCDH tetramer and SDS-PAGE for denatured GCDH isolated from liver mitochondria of 24 h fed WT and Sirt5-/- (SIRT5KO) mice. Fed Gcdh-/- (GCDHKO) mouse liver mitochondrial lysates were used as a control to identify the correct GCDH tetramer complex. J, quantitation of GCDH tetrame [file mmc6.pdf]
