# Supplementary material for: Deglutarylation of glutaryl-CoA dehydrogenase by deacylating enzyme SIRT5 promotes lysine oxidation in mice
Source: J Biol Chem. 2022 Feb 12;298(4):101723. doi: 10.1016/j.jbc.2022.101723 (PMC8969154; doi:10.1016/j.jbc.2022.101723)
Supplement: Supplemental Figure S4 — Human liver co-expression analysis for SIRT3 and 4.A and B, human liver SIRT3 liver co-expression analysis showing gene expressions that positively (A) or negatively (B) correlate to SIRT3 gene expression. C and D, human liver SIRT4 liver co-expression analysis showing gene expressions that positively (C) or negatively (D) correlate to SIRT4 gene expression. Genes highlighted in red have known roles in amino acid metabolism. [file mmc7.pdf]

Figure S4:

A

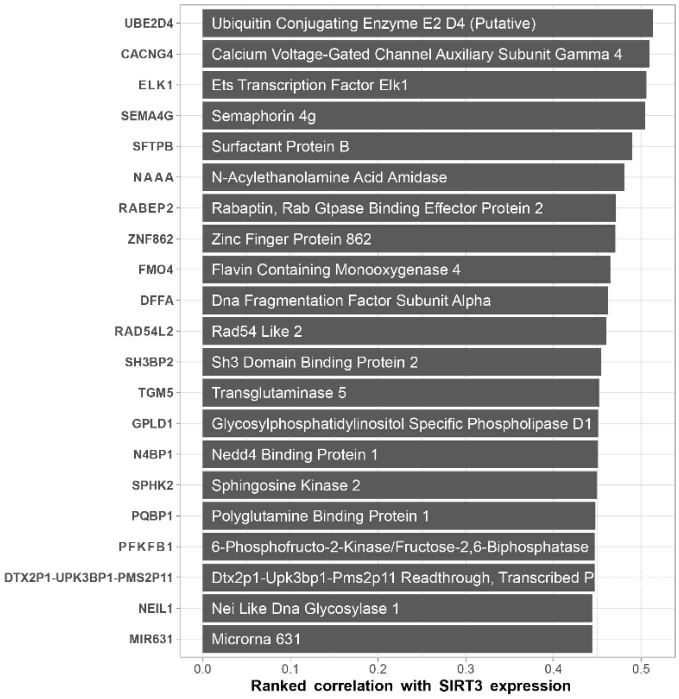

Top 20 human liver genes positively correlated with *SIRT3* expression

B

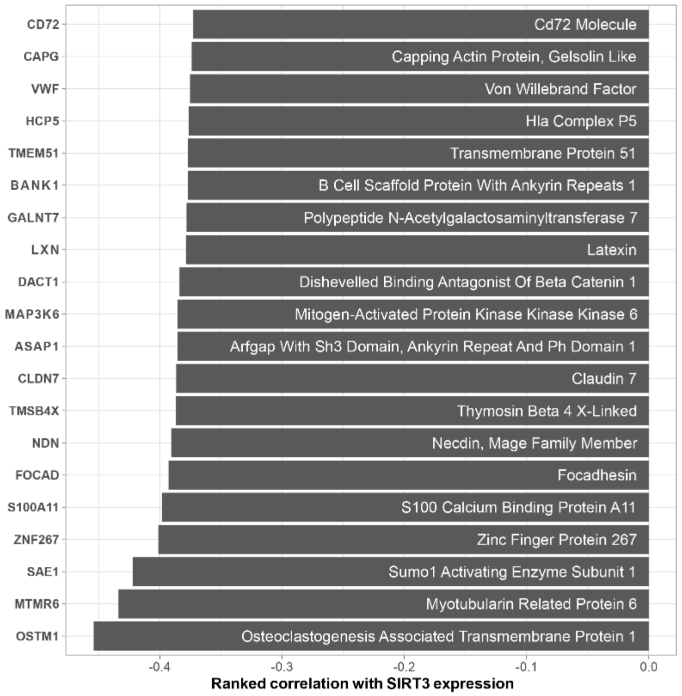

Bottom 20 human liver genes negatively correlated with *SIRT3* expression

C

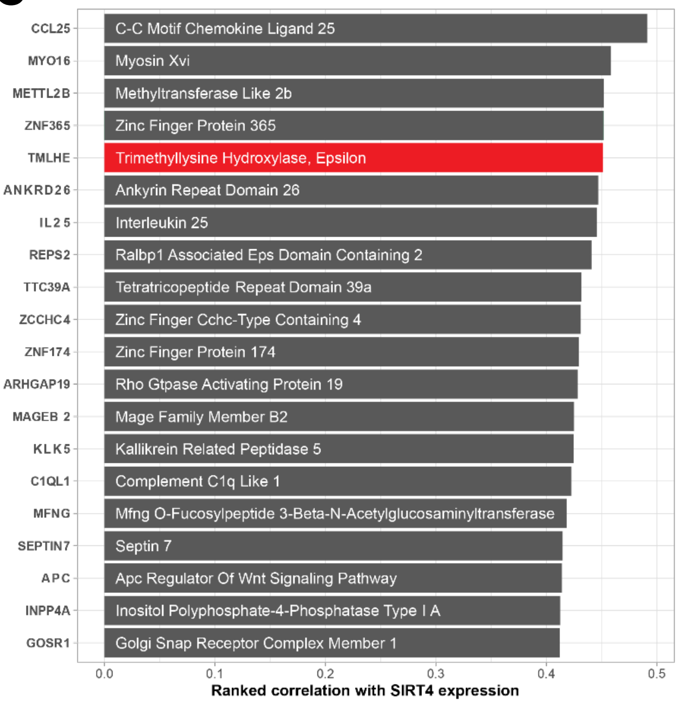

Top 20 human liver genes positively correlated with *SIRT4* expression

D

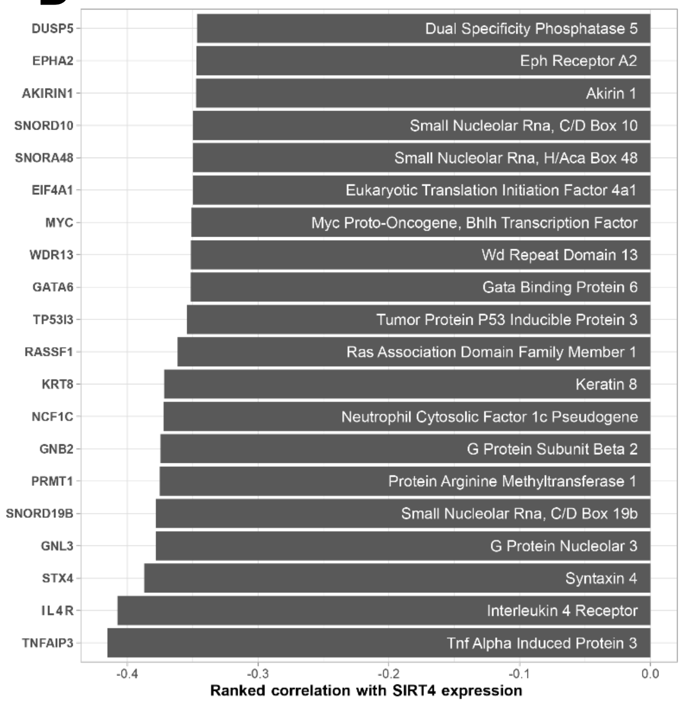

Bottom 20 human liver genes negatively correlated with *SIRT4* expression
